# Controlled Inflammation Drives Neutrophil‐Mediated Precision Drug Delivery in Heterogeneous Tumors

**DOI:** 10.1002/advs.202411307

**Published:** 2025-01-12

**Authors:** Yunfei Guo, Yiming Li, Jianmin Li, Haoran Cai, Kangkang Liu, Dengyi Duan, Wenyi Zhang, Gang Han, Yang Zhao

**Affiliations:** ^1^ Department of Radiology The Second Hospital of Tianjin Medical University Tianjin 300211 P. R. China; ^2^ Tianjin Institute of Urology The Second Hospital of Tianjin Medical University Tianjin 300211 P. R. China; ^3^ Biochemistry and Molecular Biotechnology University of Massachusetts Chan Medical School Worcester MA 01605 USA

**Keywords:** inflammation, nanomedicine, Neutrophil, tumor targeted therapy

## Abstract

Tumor heterogeneity remains a formidable obstacle in targeted cancer therapy, often leading to suboptimal treatment outcomes. This study presents an innovative approach that harnesses controlled inflammation to guide neutrophil‐mediated drug delivery, effectively overcoming the limitations imposed by tumor heterogeneity. By inducing localized inflammation within tumors using lipopolysaccharide, it significantly amplify the recruitment of drug‐laden neutrophils to tumor sites, irrespective of specific tumor markers. This strategy not only enhances targeted drug delivery but also triggers the release of neutrophil extracellular traps, further potentiating the anti‐tumor effect. Crucially, this study demonstrates that potential systemic inflammatory responses can be effectively mitigated through neutrophil transfusion, ensuring the safety and clinical viability of this approach. In a murine breast cancer model, the method significantly impedes tumor growth compared to conventional treatments. This work offers a versatile strategy for precise drug delivery across diverse tumor types. The findings pave the way for more effective and broadly applicable cancer treatments, potentially addressing the long‐standing challenge of tumor heterogeneity.

## Graphical abstract

Controlled inflammation enhances neutrophil‐mediated drug delivery to overcome tumor heterogeneity. Utilizing lipopolysaccharide (LPS) for localized inflammation amplifies neutrophil recruitment to tumor sites, allowing for precise drug release and effective tumor suppression. This innovative approach offers a safe, versatile treatment strategy applicable to diverse cancers, addressing the long‐standing challenges of tumor heterogeneity and targeted drug delivery.

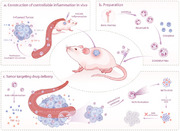

## 1 Introduction

Cancer treatment faces a persistent challenge: tumor heterogeneity. This inherent variability within and across tumor types significantly hinders the development of effective targeted therapies.^[^ ^1^ *^‐^* ^6^ ^]^ Traditional chemotherapy, while widely used, often results in poor specificity and systemic toxicity.^[^ ^7^ *^‐^* ^9^ ^]^ Recent advances in targeted therapy have improved treatment precision,^[^ ^10^ *^‐^* ^12^ ^]^ but their efficacy is limited by the diverse molecular characteristics exhibited by different patients and tumor types.^[^ ^13^ *^‐^* ^16^ ^]^

The tumor microenvironment, particularly its inflammatory component, has emerged as a promising focus for innovative cancer treatment strategies.^[^ ^17^ *^‐^* ^19^ ^]^ Neutrophils, with their inherent inflammatory chemotaxis, show considerable potential as drug carriers in tumor‐targeted therapies.^[^ ^20^ *^‐^* ^22^ ^]^ Their ability to navigate complex biological barriers and accumulate at inflammatory sites offers a natural mechanism for targeted drug delivery.^[^ ^23^ *^,^* ^24^ ^]^ Current research on neutrophil‐mediated drug delivery is primarily focused on the treatment of inflammatory diseases and postoperative recurrence of tumors.^[^ ^25^ *^,^* ^26^ ^]^ In primary tumors, the strategy typically leverages the active extravasation of neutrophils into tumor blood vessels and their penetration through tissues for targeted drug delivery.^[^ ^27^ ^]^ However, primary tumors critically lack the robust inflammatory signals needed to recruit sufficient neutrophils, resulting in inadequate drug accumulation and profoundly limiting therapeutic efficacy.^[^ ^28^ ^]^ This failure to attract neutrophils represents a major obstacle in harnessing the innate targeting capabilities of these cells for cancer treatment. The challenge is further compounded by tumor heterogeneity, which introduces variability in the inflammatory profiles across different tumor types and patients, making it difficult to achieve consistent and effective neutrophil‐mediated drug delivery.

**Scheme 1:**
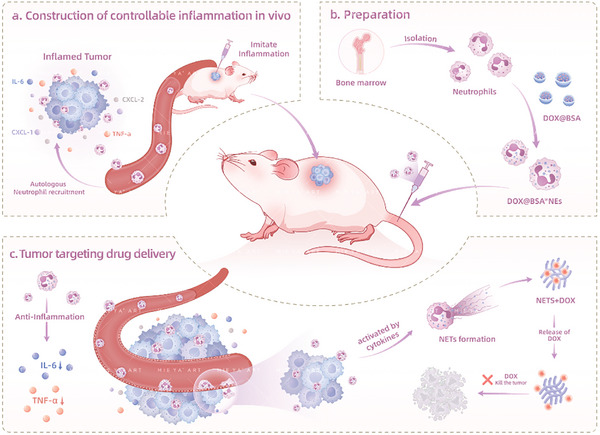
Controllable inflammation‐mediated drug delivery for tumor‐targeted therapy. a) Imitating an acute inflammatory response within the tumor leads to an elevation in the expression levels of inflammatory cytokines and chemokines. In response to the inflammatory chemotaxis, significant accumulation of autologous neutrophils occurs within the tumor in mice. b) Preparation of DOX@BSA^in^NEs. c) Schematic illustrating the recruitment of DOX@BSA^in^NEs to the interior of the tumor through the chemotactic effect of the inflammatory response for targeted treatment of the tumor.

In this study, we introduce a novel, controllable, inflammation‐mediated tumor‐targeting system that utilizes neutrophils as drug‐delivery vehicles, designed to navigate the complexity of tumor heterogeneity without relying on tumor‐specific markers. Our approach combines engineered neutrophils loaded with doxorubicin‐encapsulated bovine serum albumin nanoparticles (D‐NEs) and targeted administration of lipopolysaccharide (LPS) to induce localized inflammation within tumors (**Scheme** **1**). This strategy significantly amplifies the recruitment of drug‐laden neutrophils to tumor sites, irrespective of specific tumor markers or inherent inflammatory states, and activates the immune microenvironment within the tumor tissue, resulting in a more potent therapeutic effect.

Crucially, we demonstrate that this approach not only enhances targeted drug delivery but also triggers the release of neutrophil extracellular traps (NETs), further potentiating the anti‐tumor effect. The dual properties of neutrophils—inflammatory chemotaxis and anti‐inflammatory effects—allow for effective reduction of serum cytokine levels while exerting potent antitumor effects. Importantly, we show that potential systemic inflammatory responses can be effectively mitigated through neutrophil transfusion, ensuring the safety and clinical viability of this approach.

This work addresses a critical gap in current cancer treatment approaches, providing a solution that is both highly targeted and broadly applicable. By leveraging and enhancing the body's own immune response mechanisms, we pave the way for more effective and less toxic cancer treatments. Our approach has the potential to address the long‐standing challenges of tumor heterogeneity and insufficient neutrophil recruitment, and holds promise as a versatile oncology treatment option.

## 2 Results

### 2.1 Establishment of Controllable Inflammation

Bacterial infection is the predominant cause of clinical mastitis, and LPS—an exclusive component of the Gram‐negative bacterial cell wall—plays a pivotal role in the pathogenesis of this condition.^[^ ^29^ *^,^* ^30^ ^]^ Moreover, LPS can robustly aggregate immune cells, stimulate the release of an array of inflammatory cytokines, and modulate the immune function of the organism.^[^ ^31^ *^,^* ^32^ ^]^ Neutrophils, as the first responders of the immune system, typically mount rapid responses to all diseases in an inflammatory environment.^[^ ^33^ *^,^* ^34^ ^]^ Based on this, we envisaged injecting LPS into breast cancer tumors to induce acute inflammation within the tumor and utilizing neutrophils as drug carriers to enhance the targeting ability of drugs for tumors. Therefore, we first injected LPS into 4T1 tumors.

**Figure 1:**
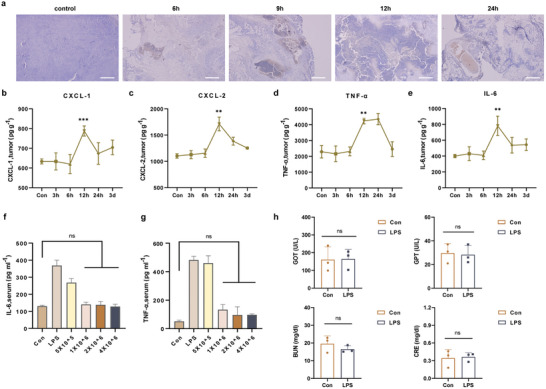
Establishment of controllable inflammation. a) Histopathology images of Ly6G‐stained tumor slices after intratumoral injection of LPS at different time points. Scale bar: 500 µm. b–e) Expression levels of CXCL‐1, CXCL‐2, TNF‐*α*, and IL‐6 in the tumor after intratumoral injection of LPS at different time points (n = 3 mice per time point). f,g). Expression levels of IL‐6 and TNF‐*α* in serum of mice treated with LPS after intravenous infusion of different amounts of neutrophils (n = 3 mice). h) Serological test results after intratumoral injection of LPS. Phosphate‐buffered saline (PBS) was used as the control (n = 3 mice). (In b–h, mean ± standard deviation (SD), ∗∗*p* < 0.01, ∗∗∗*p* < 0.001, “ns” indicates no significant difference).

To ascertain the recruitment of neutrophils induced by LPS, we injected LPS into the tumors of tumor‐bearing mice and sacrificed them at different time points to obtain tumor tissues. Immunohistochemistry (IHE) was performed to verify the accumulation of neutrophils within the tumors. The results indicated substantial aggregation of neutrophils within the tumor as early as 6 h post‐injection, with continuous accumulation observed up to 12 h and diminished accumulation after 24 h (**Figure** **1**a). To determine the optimal timing of administration for subsequent animal experiments, we employed the enzyme‐linked immunosorbent assay (ELISA) to assess the expression levels of inflammatory cytokines in tumor tissues following intratumoral injection of LPS. The results revealed remarkable increases in the expression levels of the neutrophil chemokines CXCL1 and CXCL2 within the tumor tissues after the intratumoral injection of LPS (Figure 1b,c). Additionally, the expression levels of pro‐inflammatory cytokines TNF‐*α* and IL‐6 exhibited significant increases (Figure 1d,e). Therefore, the intratumoral injection of LPS successfully induced inflammatory reactions within the tumor and promoted massive accumulation of neutrophils, which is beneficial for the precise targeted action of neutrophil cell carriers at the tumor site after intravenous administration. The expression levels of these cytokines peaked at 12 h after intratumoral injection of LPS (Figure 1b–e), and neutrophils continued to accumulate in large numbers within the tumor at 12 h after injection (Figure 1a). Therefore, the optimal time for the intravenous injection of neutrophil carriers was 12 h after the intratumoral injection of LPS.

Although LPS can induce the desired acute inflammatory response within tumors, it is crucial to prevent the systemic inflammatory reactions caused by LPS and achieve controllable inflammation. It has been reported that neutrophil transfusion can effectively suppress inflammation caused by bacterial infections in patients with neutropenia.^[^ ^35^ ^]^ Additionally, there are reports suggesting that neutrophils can reduce the toxic effects of LPS.^[^ ^36^ ^]^ Therefore, we decided to perform neutrophil transfusion in tumor‐bearing mice bearing tumors 12 h after intratumoral injection of LPS and measure the expression of inflammatory cytokines in serum to determine whether neutrophil infusion can alleviate the systemic inflammatory response induced by LPS. Intriguingly, once the quantity of neutrophils transfused reached 1 × 10*^6^* per mouse, the expression levels of pro‐inflammatory cytokines IL‐6 and TNF‐*α* in the serum returned to normal levels within 2 h after transfusion (Figure 1f,g). In contrast, in mice injected with LPS but not transfused with neutrophils, the expression levels of IL‐6 and TNF‐*α* in the serum remained high within 2 h after transfusion. Additionally, we performed hematological examinations at different time points on mice injected intratumorally with LPS. The results showed that after intratumoral injection of LPS, there was a transient increase only in the number of white blood cells and granulocytes in mice, which returned to normal levels 24 h after LPS injection (Figure S1 and Table S1, Supporting Information). These indicate that, without intervention, the inflammatory response in mice can basically return to normal 24 h after LPS injection, but transfusion of neutrophils can more effectively accelerate the recovery and alleviation of systemic inflammatory responses. To further investigate whether LPS has systemic toxic side effects, we assessed the hepatic and renal functions of tumor‐bearing mice 14 d after intratumoral injection of LPS. The results suggested that the hepatic and renal functions of tumor‐bearing mice were not compromised following the intratumoral injection of LPS (Figure 1h) and that their body weights were also unaffected (Figure S2, Supporting Information). Collectively, these findings indicate the establishment of controllable inflammation within the tumors of mice under safe and effective conditions.

### 2.2 Preparation of D‐NEs

**Figure 2:**
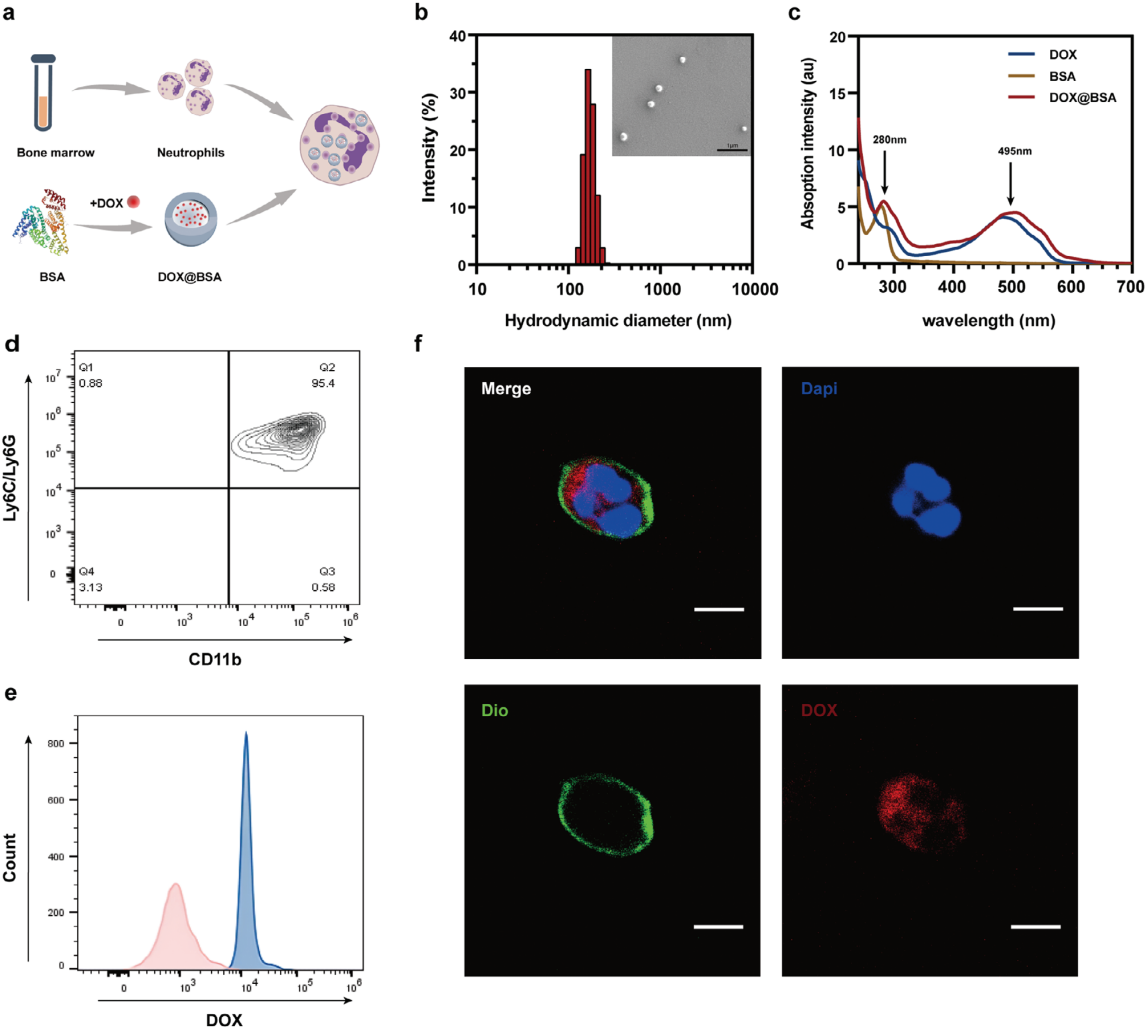
Preparation of D‐NEs. a) Schematic of the synthesis of D‐NEs. b) Particle‐size distribution of DOX@BSA measured via dynamic light scattering (DLS). Inset: SEM image of DOX@BSA. Scale bar: 2 µm. c) UV–visible (UV–vis) absorption curve of DOX@BSA. d) Flow cytometry analysis of the purity of isolated mature neutrophils with double staining of fluorescein isothiocyanate (FITC)‐conjugated anti‐mouse CD11b antibody and phycoerythrin (PE)‐conjugated anti‐mouse Ly‐6G/Ly‐6C (Gr‐1) antibody. e) Flow cytometric analysis of neutrophils cultured with a medium containing (blue) or not containing (pink) DOX@BSA for 1 h, respectively. Detection of DOX fluorescence was performed in the PE channel at 488 nm. f) CLSM images of D‐NEs. The nuclei of neutrophils were labeled with DAPI (blue), the membranes of neutrophils were labeled with Dio (green), and red fluorescence represents DOX encapsulated in the DOX@BSA. The merged image is the overlay of the three individual images. Scale bar: 5 µm.

The schematic in **Figure** **2**a illustrates the synthesis of neutrophil‐based drug carriers. In this inflammation‐mediated tumor‐targeted drug‐delivery system, we opted to extract neutrophils from mouse bone marrow and load them with nanomaterials in vitro to fabricate cellular drug carriers.^[^ ^37^ ^]^ This approach was selected over injecting nanocarriers that specifically target neutrophils in the bloodstream because it helps prevent varying levels of off‐target toxicity in healthy tissues and guarantees the administration of an adequate drug dosage in a safe and non‐immunogenic manner.

First, DOX@BSA was synthesized using the molecular switching method, with a hydrated particle size of 165 nm ± 10 nm and a negatively charged surface (Figure 2b). The spherical structure of DOX@BSA was clearly observed in scanning electron microscopy (SEM) and transmission electron microscopy images, which revealed that the average particle size was 150 nm ± 25 nm (Figure 2b; Figure S3a,b, Supporting Information). Moreover, DOX@BSA exhibited remarkable stability, maintaining a consistent particle size of ≈160 nm over 10 d (Figure S3c, Supporting Information). The ultraviolet (UV) characteristic absorption peak of DOX@BSA confirmed successful binding between DOX and BSA, with the peak intensity indicating the concentration of bound DOX in DOX@BSA (Figure 2c). Mature neutrophils were isolated from the femur and tibial bone marrow using a well‐established gradient centrifugation method. The purity of the isolated neutrophils was assessed to be >90%, according to the expression of the specific biomarkers Ly‐6G/Ly‐6C and CD11b on the membranes of mature neutrophils (Figure 2d). This confirmed the successful isolation of mature neutrophils from the mouse bone marrow. Neutrophils possess inherent phagocytic abilities that allow them to rapidly engulf exogenous nanoparticles.^[^ ^38^ ^]^ Therefore, DOX@BSA^in^Neutrophils (referred to as D‐NEs) was obtained by co‐incubating purified neutrophils with DOX@BSA, followed by collection via centrifugation. Flow cytometry was used to assess the proportion of neutrophils that engulfed DOX@BSA. The results indicated that after 1 h of co‐incubation, 99% of the neutrophils successfully engulfed DOX@BSA (Figure 2e; Figure S4a,b, Supporting Information), indicating the robust phagocytic capability of neutrophils and their potential as efficient drug carriers for tumor drug delivery. Additionally, the uptake rate of neutrophils was not significantly increased after 70 min of co‐incubation (Figure S4c, Supporting Information); thus, we selected 1 h as the co‐incubation time for preparing D‐NEs. Confocal laser scanning microscopy (CLSM) confirmed the effective engulfment of DOX@BSA by neutrophils 1 h after co‐incubation, as evidenced by the even distribution of DOX@BSA throughout the neutrophil cytoplasm (Figure 2f; Figure S5, Supporting Information). These results indicate the successful synthesis of D‐NEs.

### 2.3 Characterization of D‐NEs

**Figure 3:**
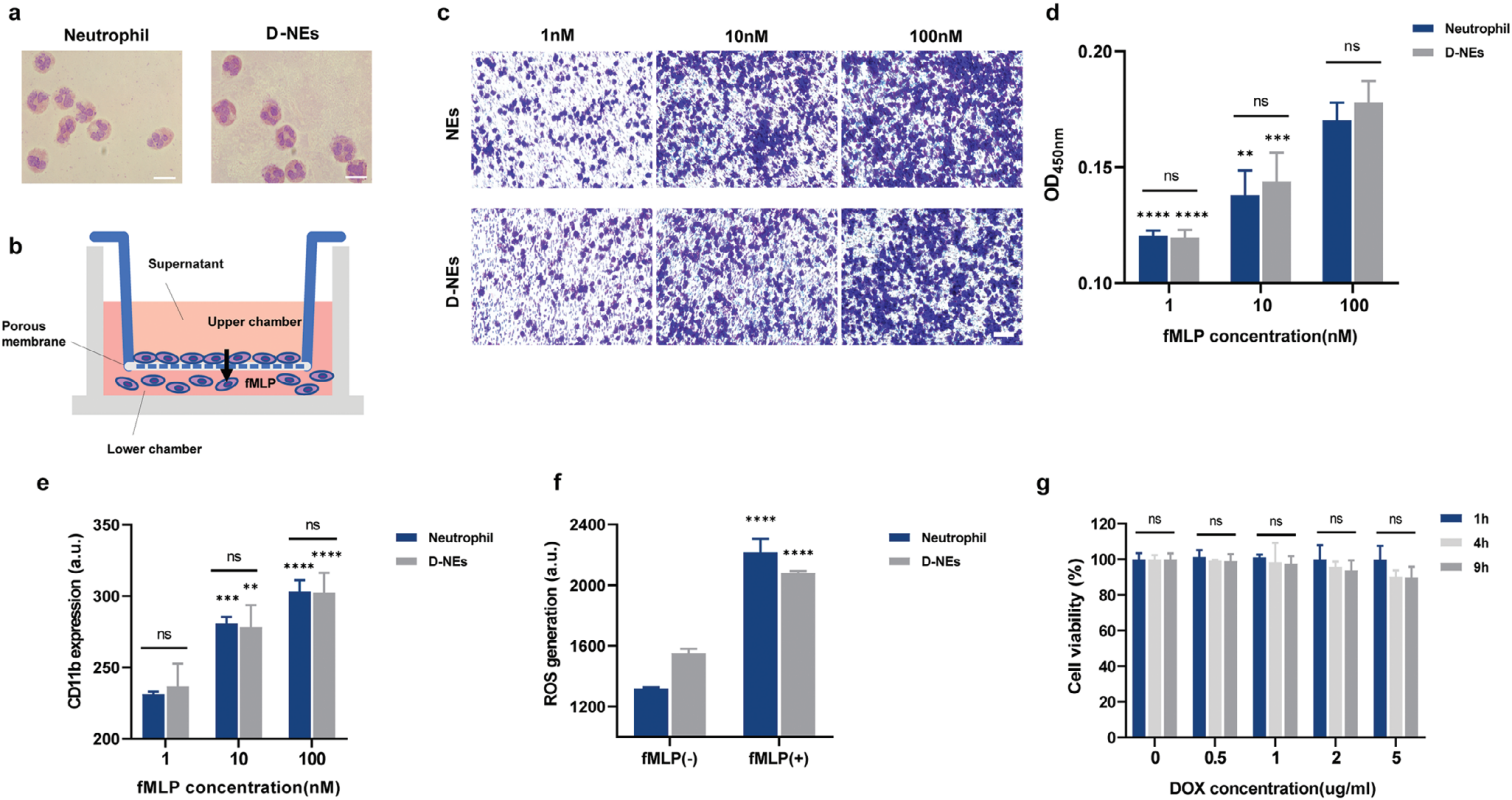
Characterization and physiological functions of D‐NEs. a) Morphological images of blank neutrophils (left) and D‐NEs (right) with Wright–Giemsa staining. Scale bar: 20 µm. b) Schematic of the in vitro migration model for determining the migration capability of D‐NEs toward inflammatory stimuli using a transwell system. c) Images of blank neutrophils and D‐NEs on the bottom side of the transwell membrane after the migration test. Scale bar: 50 µm. d) Quantification of migrated neutrophils and D‐NEs on the bottom side of the transwell membrane after the migration test. e) Flow cytometry analysis of expression levels of CD11b on cell membranes of blank neutrophils and D‐NEs after treatment with different concentrations of fMLP for 0.5 h (mean ± SD, n = 3 independent experiments). f) Flow cytometry analysis of ROS generation in blank neutrophils and D‐NEs treated with or without fMLP (1 µM) (mean ± SD, n = 3 independent experiments). g) Cell viabilities of neutrophils after incubation with DOX@BSA at different DOX concentrations for 1, 4, and 9 h. ∗*p* < 0.05, ∗∗*p* < 0.01, ∗∗∗*p* < 0.001.

Initially, we ascertained that the cellular morphology of D‐NEs remained unchanged relative to that of blank NEs via Wright–Giemsa staining, and the characteristic segmented nucleus and presence of light‐red granules within the cytoplasm were still observed (**Figure** **3**a). Subsequently, we explored the influence of nanodrug loading in the D‐NEs on specific physiological functions, including cell chemotaxis, CD11b membrane protein expression, and reactive oxygen species (ROS) production. It has been established that neutrophils actively migrate to injury or infection sites in vivo, effectively traversing the endothelial layer and infiltrating nonvascular regions guided by the concentration gradient of chemotactic factors.^[^ ^39^ ^]^ This phenomenon—termed “chemotaxis”—is a cornerstone of our inflammation‐mediated drug‐delivery system.^[^ ^40^ ^]^ Therefore, we employed the neutrophil chemotactic peptide N‐formyl‐met‐leu‐phe (fMLP) to stimulate neutrophils in vitro, thereby simulating the chemotactic process observed in vivo.^[^ ^41^ *^,^* ^42^ ^]^ Transwell migration assays were conducted to evaluate the chemotactic ability of D‐NEs (Figure 3b). Intriguingly, D‐NEs and blank NEs exhibited analogous activation characteristics in response to fMLP; therefore, loading DOX@BSA did not affect neutrophil chemotaxis (Figure 3c,d; Figure S6, Supporting Information). CD11b‐mediated neutrophil adhesion and extravasation are essential for neutrophil immune function. In response to stimuli, such as LPS, IL‐8, and fMLP, the expression of CD11b is prominently upregulated.^[^ ^43^ *^‐^* ^45^ ^]^ As expected, following fMLP treatment, CD11b expression in D‐NEs increased significantly with an increase in the fMLP concentration. Furthermore, the extent of upregulation was comparable to that observed in blank NEs (Figure 3e). According to previous reports, activated neutrophils can eradicate tumor cells by generating ROS, thereby exerting direct cytotoxic effects. Similarly, upon fMLP treatment, both the D‐NEs and blank NEs exhibited increased ROS generation (Figure 3f). These results indicate that the vital physiological functions of neutrophils remain unaffected despite the loading of DOX@BSA, providing a crucial prerequisite for successful targeted drug delivery by neutrophils.

To further ascertain the viability of neutrophils upon infusion into the body and ensure their ability to migrate to the tumor site within the optimal time window, DOX@BSA was co‐incubated with neutrophils for varying durations in vitro. Subsequently, the cytotoxicity of DOX@BSA against neutrophils was assessed using a CCK‐8 assay. The results indicated that within 9 h, DOX@BSA at the concentrations employed did not cause notable toxicity to neutrophils, with their viability remaining above 80% (Figure 3g). Compared with DOX@BSA, free DOX caused significant cytotoxicity toward neutrophils (Figure S7, Supporting Information). This substantiated our decision to incorporate a chemotherapeutic drug into neutrophils using nanocarriers, as it allows the maintenance of drug loading while circumventing the toxic effects exerted by the chemotherapeutic drug on the cell carriers.

### 2.4 In Vitro Inflammation‐Induced Drug Release and Tumoricidal Effect

**Figure 4:**
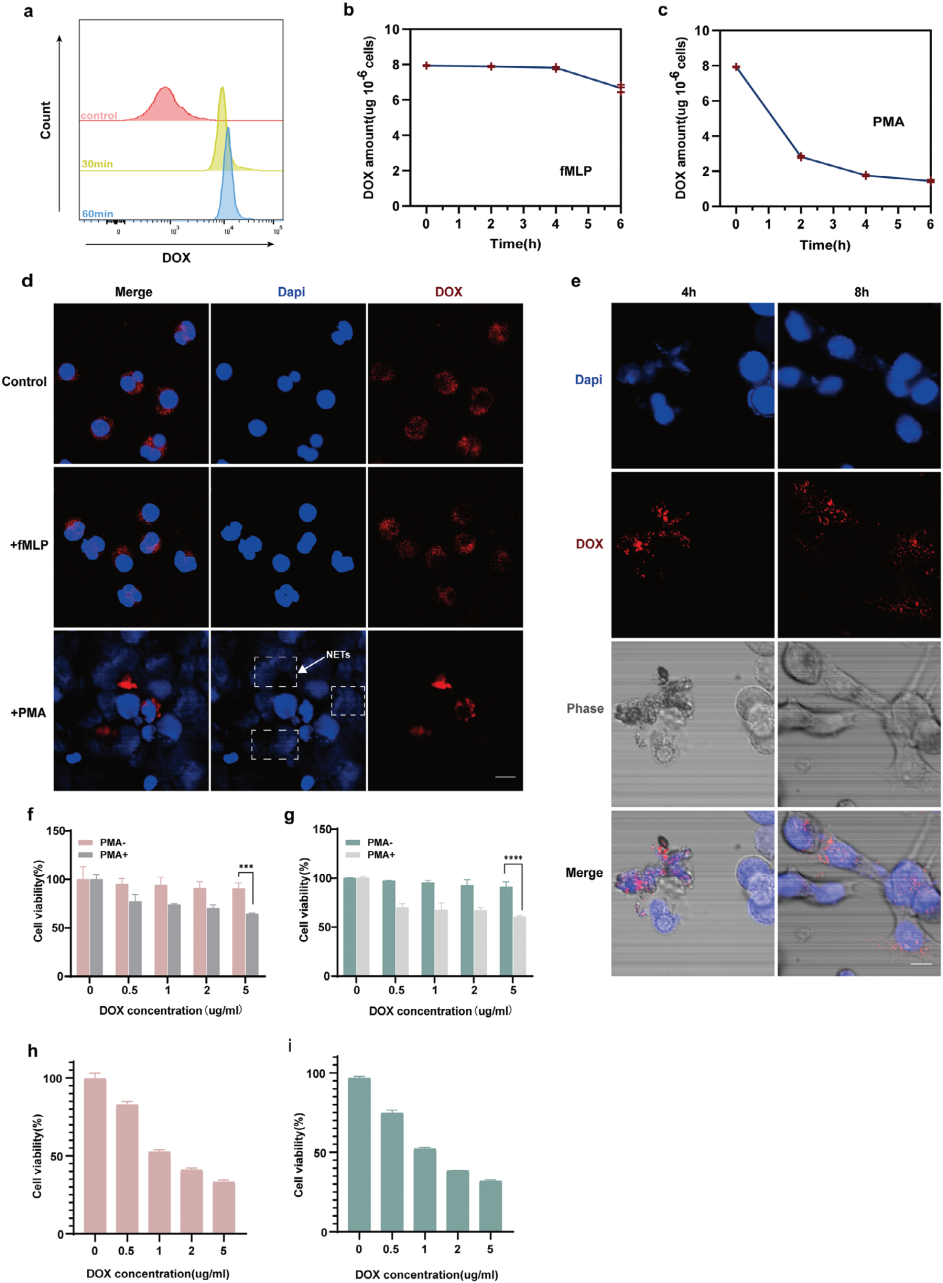
In vitro inflammation‐induced drug release and tumoricidal effect. a) Uptake of DOX@BSA by neutrophils after incubation at different time points. b,c) Determination of the amount of DOX retained in D‐NEs in the presence of fMLP (10 nM) (b) or PMA (100 nM) (c) over time (mean ± SD, n = 3 independent experiments). d) CLSM images of NETs released by D‐NEs after 6 h of treatment with fMLP and PMA. Neutrophil‐derived DNA networks were labeled with DAPI (blue), and red fluorescence represents DOX encapsulated in the DOX@BSA. Scale bar: 10 µm. e) CLSM images of PMA‐treated D‐NEs incubated with 4T1 cells for 4 and 8 h. 4T1 cell nuclei and neutrophil‐derived DNA networks were labeled with DAPI (blue), and red fluorescence represents DOX encapsulated in the DOX@BSA. Scale bar: 10 µm. f,g) Cytotoxicity of PMA‐treated D‐NEs at different DOX concentrations to 4T1 cells (left) and PC3 cells (right) (mean ± SD, n = 3 independent experiments, ∗∗∗*p* < 0.001, ∗∗∗∗*p* < 0.0001, two‐way analysis of variance (ANOVA). h,i) Cytotoxicity of LPS‐treated D‐NEs at different DOX concentrations to 4T1 cells (left) and PC3 cells (right) (n = 3).

To investigate the in vitro release of DOX@BSA from neutrophils upon inflammatory stimulation, we determined the saturation state of DOX@BSA uptake by neutrophils using flow cytometry (**Figure** **4**a). Subsequently, saturated D‐NEs were incubated in different culture media to mimic the chemotaxis of neutrophils and the site of inflammation during inflammatory events, using an RPMI‐1640 medium supplemented with fMLP or PMA.^[^ ^46^ ^]^ As expected, neutrophils cultured in the medium with fMLP exhibited an intracellular quantity of DOX@BSA comparable to that in the saturated state within 4 h, with a slight reduction observed at 6 h (Figure 4b). In contrast, neutrophils cultured in the medium with PMA exhibited an explosive release of DOX@BSA within 2 h, leading to a significant reduction in the intracellular DOX@BSA quantity, which continued to decrease with an increase in the incubation time (Figure 4c). Under an optical microscope, neutrophils treated with fMLP did not exhibit significant changes in cell morphology, and the cell membrane retained its integrity (Figure S8a, Supporting Information), whereas neutrophils treated with PMA exhibited significant alterations in cell morphology (Figure S8b, Supporting Information). The release of DOX@BSA was verified using CLSM. After 6 h of treatment with fMLP and PMA, D‐NEs produced NETs only in the presence of PMA, and DOX@BSA was visible in the NETs. (Figure 4d). Meanwhile, we also treated D‐NEs with medium containing LPS for 6 h, and D‐NEs similarly produced NETs (Figure S9a, Supporting Information). These findings indicate that neutrophils selectively release DOX@BSA in an inflammatory environment while maintaining cell integrity during chemotaxis, thereby preventing the premature leakage of DOX@BSA. Regarding the form of doxorubicin released from neutrophils, we conducted further experiments and found that it remains in the form of BSA‐encapsulated DOX after release (Figure S9b, Supporting Information). It has been reported that neutrophils can migrate to the site of inflammation within 6 h after injury, suggesting that D‐NEs can effectively achieve targeted delivery to tumors, avoiding off‐target toxicity caused by premature release during chemotaxis.^[^ ^47^ ^]^

To further explore the mechanism whereby neutrophils release DOX@BSA to kill tumor cells, we cultured 4T1 cells in confocal dishes and incubated them with PMA‐treated D‐NEs. After co‐incubation for 4 h, accompanied by NET formation, DOX@BSA was released by D‐NEs, but was not observed in 4T1 cells at this time point. With the incubation time extended to 8 h, DOX@BSA uptake by 4T1 cells was observed (Figure 4e). Similar results were also observed with D‐NEs treated with LPS (Figure S10, Supporting Information). In contrast, in the absence of PMA treatment, DOX@BSA remained in the cytoplasm of neutrophils but was absent in 4T1 cells (Figure S11, Supporting Information). These results indicate that neutrophils can release DOX@BSA under inflammatory stimulation and that DOX@BSA is subsequently taken up by tumor cells owing to its nanoscale size. We investigated the cytotoxic effects of D‐NEs on 4T1 and PC3 cells in vitro using the CCK8 assay. The PMA‐treated D‐NEs exhibited significant cytotoxic effects on both 4T1 and PC3 cells, whereas the untreated D‐NEs did not exhibit significant antiproliferative activity (Figure 4f,g; Figure S12, Supporting Information). Meanwhile, D‐NEs treated with LPS also exhibited significant cytotoxic effects on both 4T1 and PC3 cells (Figure 4h,i). We further validated the versatility of this protocol using other breast cancer and prostate cancer cell lines (Figure S13, Supporting Information). Therefore, inflammatory stimulation is crucial for the release of DOX@BSA by neutrophils, which determines whether therapeutic drugs can be taken up by tumor cells to effectively exert their antitumor effects.

### 2.5 Tumor‐Directed Chemotaxis Exhibited by D‐NEs and Accumulation of DOX Within Tumor Site In Vivo

**Figure 5:**
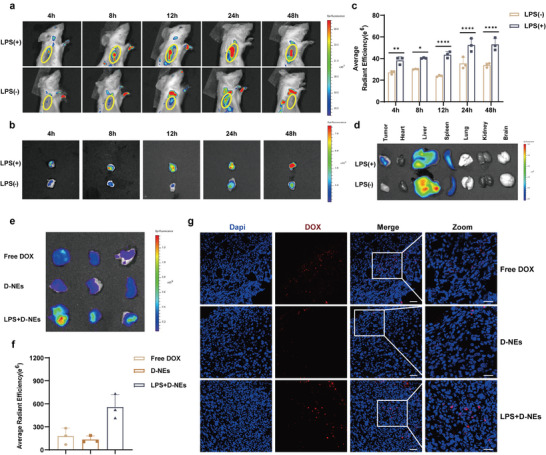
Tumor‐directed chemotaxis exhibited by D‐NEs and the accumulation of DOX within the tumor site in vivo. a) In vivo fluorescence imaging of mice after intravenous injection of DD‐NEs in non‐LPS‐treated and LPS‐treated 4T1 tumor‐bearing mice over time. b) Ex vivo fluorescence imaging of tumors excised from mice at different time points after intravenous injection of DD‐NEs in untreated and LPS‐treated 4T1 tumor‐bearing mice. c) Quantification analysis of the relative fluorescence intensity in the tumors at different time points (mean ± SD, n = 3, ∗*p* < 0.05, ∗∗*p* < 0.01, ∗∗∗*p* < 0.001, ∗∗∗∗*p* < 0.0001, two‐way ANOVA). d) Ex vivo fluorescence imaging of the major organs. e) Ex vivo fluorescence imaging of DOX in excised tumors from mice 24 h after different treatments and f) the corresponding semiquantitative analysis of (e) (mean ± SD, n = 3). g) CLSM images of tumor slices excised from mice 24 h after different treatments. The nuclei of tumor cells were labeled with DAPI (blue), and red fluorescence represents DOX. Scale bar: 30 µm.

The tumor‐targeting effect of D‐NEs in vivo was verified using an In Vivo Imaging System (IVIS Spectrum) in a breast cancer tumor model with controllable inflammation. Initially, neutrophils were labeled with the cell membrane fluorescent dye DiD and co‐incubated with DOX@BSA in vitro, resulting in the formation of DiD‐labeled D‐NEs (DD‐NEs). Following intravenous injection, DD‐NEs exhibited significantly enhanced tumor‐targeting capability in breast cancer mice with controllable inflammation relative to untreated breast cancer mice, and strong fluorescence was observed at the tumor site as early as 4 h post‐injection. The fluorescence signal at the tumor site exhibited a sustained high intensity exceeding that for untreated breast cancer mice for up to 48 h post‐injection (Figure 5a,b). Quantification of the region of interest (ROI) confirmed these results (**Figure** **5**c). To image and quantify the fluorescence signals in the main organs, tumor‐bearing mice were sacrificed 12 h post‐injection, and the harvested organs were imaged (Figure 5d). The results revealed that in untreated breast cancer mice, DD‐NEs were mainly distributed in the liver and spleen. However, in breast cancer mice with controllable inflammation, compared with the control group, a significant reduction in the extent of DD‐NE accumulation within the liver was observed, while increased accumulation was detected within the tumor tissue. These findings indicate the importance of inducing controllable inflammation within the tumor, which effectively amplifies inflammatory signals and significantly enhances the tumor‐targeting capability of neutrophils.

To determine the accumulation of the chemotherapeutic drug DOX within the tumor site following the establishment of controllable inflammation, we employed tumor‐bearing mice injected with free DOX and untreated breast cancer mice as control groups. Notably, tumor‐bearing mice injected with free DOX were also subjected to the induction of controllable inflammation within the tumor. After 24 h of treatment, the three groups of tumor‐bearing mice were sacrificed, and the tumor tissues were collected for imaging. Relative to the mice injected with free DOX and the mice with untreated breast cancer, mice with controllable inflammation utilizing neutrophil‐mediated delivery exhibited a higher accumulation of DOX in the tumor, with a stronger fluorescence signal observed at the tumor site (Figure 5e), as confirmed by quantitative ROI analysis (Figure 5f). Additionally, we performed immunofluorescence staining of the collected tumor tissues to demonstrate the targeted delivery of D‐NEs mediated by the inflammatory response. CLSM exhibited the strongest DOX fluorescence of D‐NEs within the tumors of breast cancer mice with controllable inflammation, which indicated a large infiltration into the tumor tissue (Figure 5g; Figure S14, Supporting Information). This confirmed that the D‐NEs effectively delivered DOX to the interior of the tumor, exhibiting a superior tumor‐targeting ability under the mediation of the inflammatory response.

### 2.6 Antitumor Effect In Vivo Mediated by Inflammation Within Tumor

**Figure 6:**
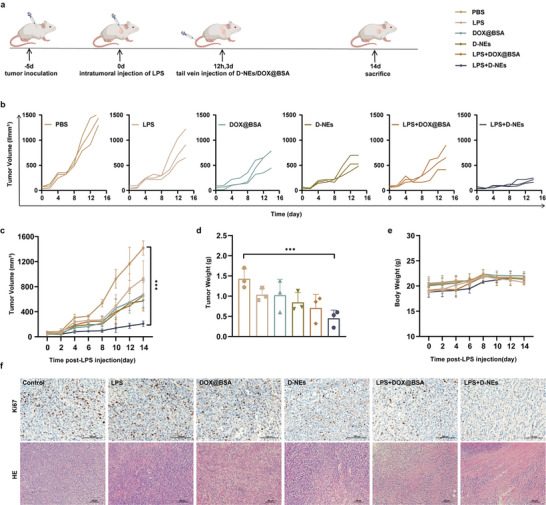
Antitumor effect in vivo mediated by inflammation within tumor. a) Schematic of the therapeutic schedule for the treatment of 4T1 tumors by the inflammation‐mediated neutrophil drug‐delivery system. 4T1 tumor‐bearing mice were treated with (+) or without (−) LPS and subsequently received different formulations of saline, DOX@BSA, or D‐NEs at hour 12 and day 3. The mice were sacrificed on day 14, and tumor tissues were collected. b,c) Relative tumor volumes with different treatments from day 0 to day 14 (mean ± SD, ∗∗∗*p* < 0.001). d) Weights of the final tumors with different treatments (mean ± SD, ∗∗*p* < 0.01). e) Body weights of mice in different treatment groups (mean ± SD). f) Hematoxylin and eosin (H&E) staining and Ki67 staining of tumor slices.

To evaluate the antitumor efficacy of the D‐NEs, we established a 4T1 heterotopic breast cancer tumor model in BALB/c mice. According to the in vivo therapeutic schedule, mice were randomly divided into six groups, with three groups undergoing the induction of controllable inflammation within the tumor via intratumoral injection of LPS, while the other three groups remained untreated. The three groups without controllable inflammation within the tumor were administered intravenous saline (control group), DOX@BSA, or D‐NEs. Similarly, the three groups with controllable inflammation within the tumor were administered intravenous saline, DOX@BSA, and D‐NEs (**Figure** **6**a). The tumor volume was monitored and measured daily. As shown in Figure 6b–d and Figure S15 (Supporting Information), the tumors in the control group exhibited rapid growth within 14 d. In non‐LPS‐treated tumor models, D‐NEs exhibited limited effectiveness in inhibiting tumor growth; however, in LPS‐treated tumor models, D‐NEs significantly slowed tumor growth and demonstrated superior antitumor toxicity compared to all other experimental groups. Tumor cell morphology was further assessed by histological analysis. Remarkably, the last group exhibited a substantial number of tumor cell deaths and the lowest levels of Ki67 expression compared to the other groups (Figure 6f). This implies that the establishment of inflammation within the tumor remarkably amplifies the tumor‐targeting proficiency of neutrophils and promotes the accumulation of DOX within the tumor.

Additionally, LPS has been reported that LPS has immunostimulatory effects.^[^ ^48^ ^]^ Therefore, to assess the impact of LPS treatment on the immune response of mice, we performed immunofluorescence staining of tumor tissues to detect the presence of CD4^+^ and CD8^+^ T cells within the tumor tissues. The results showed that the number of CD4^+^ and CD8^+^ T cells within the tumor tissue of mice treated with LPS was higher than that in the untreated tumor tissue (Figures S16 and S17, Supporting Information), indicating the immunostimulatory effect of LPS. The proportion of mature dendritic cells (DCs) and M1 macrophages in tumor tissues has also increased significantly (Figure S18, Supporting Information).

The biosafety of LPS and D‐NEs was jointly evaluated by continuously monitoring body weight and examining pathological changes in the major organs of the mice. Notably, no significant differences in body weight were observed between any of the experimental and control groups (Figure 6e). Furthermore, histological analysis of major organ sections obtained on day 14 revealed no notable abnormalities or organ damage in the heart, liver, spleen, lungs, or kidneys of mice treated with LPS and D‐NEs (Figure S19, Supporting Information). There was no impairment in liver or kidney function (Figure S20, Supporting Information). These results collectively confirmed the excellent biocompatibility of LPS and D‐NEs.

## 3 Discussion

Cancer treatment faces several challenges. Traditional chemotherapy, which is widely used in clinical practice, has poor specificity and systemic toxicity.^[^ ^49^ ^]^ Additionally, some patients develop resistance to chemotherapy, limiting the sustained efficacy of treatment.^[^ ^50^ ^]^ In recent years, targeted therapy has advanced rapidly as a treatment strategy that relies on precise interactions with specific molecular targets in tumor cells or the microenvironment.^[^ ^51^ *^,^* ^52^ ^]^ Compared with conventional cancer treatment methods, targeted therapy enhances treatment specificity, alleviates the burden of severe side effects, and improves treatment precision. However, this method has inherent limitations. Currently, most targeted therapies focus on precise targeting of individual molecules associated with a specific type of cancer. Owing to the heterogeneity of tumors, individuals and various types of cancers may exhibit distinct molecular characteristics, leading to variable treatment responses for the same targeted approach.^[^ ^53^ ^]^ Moreover, the lack of a comprehensive targeted strategy hinders the ability to address the diversity of patients and tumor types.^[^ ^54^ ^]^ Consequently, targeted therapy currently serves merely as a supplementary approach to clinical cancer treatment.

Recently, harnessing the characteristics of the inflammatory microenvironment has emerged as a focal point in the development of innovative cancer treatment strategies.^[^ ^55^ *^,^* ^56^ ^]^ Owing to their inherent inflammatory chemotaxis, neutrophils have considerable potential as drug carriers in tumor‐targeted therapies.^[^ ^57^ ^]^ They exhibit high specificity, allowing targeted delivery to inflammatory sites and thereby enhancing the precision of drug delivery. Moreover, neutrophils are abundantly recruited during inflammatory responses and serve as natural and rich resources for drug carriers. Additionally, neutrophils can release drug molecules that exert therapeutic effects on tumors.^[^ ^58^ *^‐^* ^60^ ^]^ This multifaceted approach capitalizes on the innate properties of neutrophils and is a promising avenue for advancing targeted and effective cancer therapies. Therefore, exploring the optimization of the drug‐carrier role of neutrophils in a tumor inflammatory model is paramount for the development of innovative cancer treatment methods. In this study, we developed an inflammation‐mediated neutrophil drug‐delivery system that induces controllable inflammation within tumor tissues.

By ingeniously leveraging the dual characteristics of neutrophils, which can both target and counteract inflammation, we developed a harmless approach for the precise and effective targeted delivery of antitumor drugs to enhance antitumor efficiency. In contrast to the focus on singular tumor‐specific markers, neutrophil‐based nanoplatforms exhibit heightened targeting capabilities by eliciting an amplified inflammatory response within the tumor microenvironment. Neutrophil shells maintain structural and functional integrity during chemotaxis and migrate extensively to the tumor under the influence of chemokines, such as CXCL1 and CXCL2.^[^ ^61^ ^]^ Stimulated by the inflammatory environment within the tumor, neutrophils release NETs, facilitating substantial accumulation of the chemotherapeutic drug DOX at the tumor site.^[^ ^62^ ^]^ This approach exhibited outstanding therapeutic efficacy in a murine model of breast cancer, significantly delaying tumor growth. While there may be a transient elevation in circulating inflammatory cytokines and other indicators due to the potential leakage of LPS into the bloodstream, prompt control of inflammation is evident upon neutrophil infusion, resulting in the normalization of all parameters.

The study has several limitations that should be acknowledged. First, the current approach requires loading the drug into neutrophils in vitro and then injecting them back into the body, which may not be the most practical or efficient method for clinical applications. Although it has been reported that neutrophilic drug carriers have been synthesized in vivo,^[^ ^63^ ^]^ targeting the drug to neutrophils in vivo may still present challenges and could result in off‐target effects. Additionally, while the study demonstrates the efficacy of the neutrophil drug‐delivery system in a murine model of breast cancer, further validation is needed to establish its broad‐spectrum applicability across different tumor types. Testing the approach in various tumor models would provide more robust evidence of its effectiveness and potential utility in clinical settings. Moving forward, it is essential to explore simpler and safer neutrophil drug delivery strategies that can be easily translated into clinical practice. This could involve further optimizing the drug‐loading process or exploring alternative methods for targeting neutrophils in vivo.

By integrating inflammation into cancer treatment and leveraging immune cells as drug carriers, we optimized the targeted delivery of chemotherapeutic drugs in a safe and non‐immunogenic manner. This camouflaging strategy can also be applied to the construction of other biological hybrid nanoplatforms by integrating chemotherapeutic drugs or functional nanoparticles into living cells. This innovative approach overcomes critical challenges associated with current clinical cancer therapies, such as poor specificity and high systemic toxicity. Importantly, the proposed drug‐delivery system has the potential to disregard the impact of tumor heterogeneity and achieve broad‐spectrum treatment across various types of tumors.

## 4 Experimental Section

### Preparation and Characterization of DOX@BSA

DOX‐loaded BSA nanoparticles were prepared via a molecular switching process, as previously reported. First, 10 mg of BSA was added to 5 mL of deionized water to obtain a BSA aqueous solution. The solution was poured into a round‐bottom flask with continuous stirring at 37 °C and 500 rpm, and then 35 µL of *β*‐mercaptoethanol was added, to reversibly open the disulfide bond in BSA. Three minutes later, an NaOH solution with a molar concentration of 0.1 M was slowly added to the flask, adjusting the pH of the solution to 10. Subsequently, it weighed 1.5 mg of DOX·HCl and dissolved it in 500 µL of deionized water to prepare a 3 mg mL^−1^ DOX·HCl aqueous solution. The DOX·HCl aqueous solution (3 mg mL^−1^) was slowly added to the aforementioned solution under stirring and continued stirring at 37 °C for 0.5 h. Subsequently, the obtained solution was centrifuged at 10000 rpm for 10 min, and the supernatant was collected. The final product—DOX@BSA nanoparticles—was obtained after ultrafiltration thrice with a 30‐kD ultrafiltration tube at 5000 rpm.

The particle size was characterized via DLS (NanoZS90; Malvern, UK). For SEM (Gemini300T; ZEISS, Germany), DOX@BSA was dropped onto a silicon wafer (3 mm^2^). After air‐drying, the silicon wafer was glued onto conductive carbon tape. Under vacuum, gold particles were deposited on the samples using a sputtering coater. SEM imaging was performed at an accelerating voltage of 2 kV. UV–vis spectroscopy was used to measure the DOX concentration at a wavelength of 490 nm. The encapsulation efficiency (EE% (W_1_/W_2_ × 100%) was calculated, where W_1_ and W_2_ represent the amounts of DOX in DOX@BSA before and after ultrafiltration, respectively.

### Isolation and Characterization of Murine Mature Neutrophils from Bone Marrow

Mature murine neutrophils were isolated from mouse bone marrow using a neutrophil isolation kit (Solarbio). Briefly, the femur and tibia of mice were first stripped and soaked in 75% alcohol for 3 min, and then the bone marrow in the femur and tibia was gently flushed out with a tissue diluent. This procedure was repeated until the bone marrow was completely washed, centrifuged at 250 g for 5 min, and resuspended in PBS. The unicellular suspension was carefully spread into a centrifuge tube containing a neutrophil separation solution and centrifuged for 0.5 h at 1000g. Then, the lower annular milky white cell layer was carefully sucked into a 15‐mL centrifuge tube, added a cell washing solution to 10 mL, performed centrifugation for 10 min at 250 g, discarded the supernatant, and washed the cell precipitate with a cell washing solution thrice to obtain pure mature neutrophils (NEs). NEs were resuspended in PBS for subsequent experiments. The purity of the obtained mature NEs was determined via flow cytometry staining with FITC‐conjugated anti‐mouse CD11b antibody (50 µg mL^−1^, Thermo Fisher) and PE‐conjugated anti‐mouse Ly‐6G/Ly‐6C (Gr‐1) antibody (0.2 mg mL^−1^, Thermo Fisher).

### Loading Nanoparticles into Neutrophils and Characterization of D‐NEs

Nanoparticle loading was achieved by incubating DOX@BSA with neutrophils. First, isolated mature neutrophils were seeded in sterile tubes containing a fetal bovine serum (FBS)‐free RPMI‐1640 medium for 1 h. This was followed by incubation with DOX@BSA at a DOX concentration of 5 µg mL^−1^ at 37 °C for 1 h. After centrifugation and washing thrice with PBS to remove the uninternalized nanoparticles, pure D‐NEs were obtained and resuspended in PBS for subsequent experiments. The morphology of the D‐NEs was examined using Wright–Giemsa staining. The uptake efficiency of DOX@BSA by neutrophils was measured using flow cytometry, and the location of DOX@BSA within neutrophils was examined using CLSM. To determine the quantity of DOX in neutrophils, the D‐NEs were disrupted using a cell lysis buffer (Beyotime) to release DOX from the NEs. The cell lysate was collected and centrifuged at 10000 g for 5 min. The absorbance of the supernatant was measured using a fluorescence spectrophotometer (FL).

### Assessment of Cytotoxicity of DOX@BSA Against Neutrophils

The cytotoxicity of DOX@BSA against neutrophils was measured using Cell Counting Kit‐8 (CCK8). Neutrophils were seeded in 96‐well plates at a density of 3 × 10^4^ cells/well and incubated with an FBS‐free RPMI‐1640 medium for 1 h. Next, the NEs were cultured with DOX@BSA at different DOX concentrations in 5% CO_2_ at 37 °C for 1, 2, and 9 h. At the end of the incubation, 10 µL of the CCK8 solution was added to each well of the plate, followed by further incubation for 90 min. The absorbance was measured at a wavelength of 450 nm using a microplate reader. The relative cell viability was calculated as (At − Anc)/(Apc − Anc) × 100%, where At, Apc, and Anc represent the absorbances of the tested groups and positive and negative controls, respectively.

### Evaluation of Physiological Functions of D‐NEs

To achieve inflammation‐mediated drug delivery, the physiological functions of the D‐NEs were evaluated, including CD11b protein expression on the neutrophil cell surface, chemotaxis, and ROS. The inflammation‐responsive chemotaxis of D‐NEs was determined in vitro a transwell cell migration assay (polycarbonate membrane, pore size: 3 µm, membrane diameter: 6.5 mm, surface area: 0.33 cm^2^, Corning). Briefly, blank neutrophils and D‐NEs (2 × 10^5^ cells) were added to the upper chamber of the Transwell plate, and the wells of the plate (lower chambers) were filled with an FBS‐free 1640 medium containing different concentrations of N‐formylmethionyl‐leucyl‐phenylalanine (fMLP,1 nM and 100 nM). After 90 min of incubation, the cell culture inserts were removed, and the cells in the lower chambers were counted using an optical microscope.

The expression level of CD11b on the cell membrane was investigated via flow cytometry. Blank NEs and D‐NEs were treated with different concentrations of fMLP at 37 °C for 0.5 h. After washing with ice‐cold PBS thrice, FITC‐conjugated CD11b antibody (50 µg mL^−1^, Thermo Fisher) was added to conjugate to protein for 30 min, followed by washing with PBS thrice. The fluorescence intensity was determined using flow cytometry (ACEA NovoCyte, SV, USA).

The inflammation‐mediated ROS‐generating capability of the D‐NEs was examined using 2′,7′‐dichlorodihydrofluorescein diacetate (DCFH‐DA). The blank neutrophils and D‐NEs were treated with fMLP (1 µM) at 37 °C for 0.5 h, followed by washing with ice‐cold PBS thrice, and then stained with DCFH‐DA at 37 °C for 0.5 h. After centrifugation and washing with PBS thrice, the fluorescence intensity was measured using flow cytometry (ACEA NovoCyte, SV, USA).

### Release of DOX@BSA from D‐NEs Under Different Conditions

The stimulated release of DOX@BSA from neutrophils was determined under different conditions, including the chemotactic response to local inflammation and at the site of inflammation. fMLP was used to simulate chemotactic cytokines in blood circulation, and PMA was used to simulate inflammatory sites. Briefly, D‐NEs (1 × 10^6^ cells/well) were seeded in six‐well plates, and the wells of the plate were filled with an FBS‐free RPMI 1640 medium containing fMLP (10 nM) and PMA (100 nM). The D‐NEs were then incubated for different periods (0, 2, 4, and 6 h). The amount of DOX in the NEs was measured using an FL.

NET formation in NEs induced by PMA was observed using CLSM (FLUOVIEW FV1000, Olympus). Briefly, D‐NEs (1 × 10^6^ cells/well) were seeded in confocal dishes and then incubated with an FBS‐free 1640 medium containing fMLP (10 nM) or PMA (100 nM) in 5% CO_2_ at 37 °C for 0 and 6 h. Subsequently, 4% PFA was used to fix the samples, and the released DNA fragments and cell nuclei were stained with DAPI for 5 min. After washing twice with ice‐cold PBS, the cells were visualized using CLSM (FLUOVIEW FV1000; Olympus).

NET formation in NEs induced by LPS was observed using CLSM (FLUOVIEW FV1000, Olympus). To observe the release form of DOX@BSA, BSA was labeled with FITC for subsequent experiments. Briefly, D‐NEs (1 × 10^6^ cells/well) were seeded in confocal dishes and then incubated with an FBS‐free 1640 medium containing LPS(5µg mL^−1^) in 5% CO_2_ at 37 °C for 0 and 6 h. Subsequently, 4% PFA was used to fix the samples, and the released DNA fragments and cell nuclei were stained with DAPI for 5 min. After washing twice with ice‐cold PBS, the cells were visualized using CLSM (FLUOVIEW FV1000; Olympus).

### Cell Culture

Mouse breast cancer (4T1) and human breast cancer (MB231) and human prostate cancer (PC3, DU145, C42) cell lines were used in this study. Cells(4T1, PC3, DU145, C42) were maintained in a 1640 medium supplemented with FBS (10%, v:v), penicillin (100 U/mL), and streptomycin (100 µg/mL). Cells(MB231) were maintained in a DMEM medium supplemented with FBS (10%, v:v), penicillin (100 U/mL), and streptomycin (100 µg/mL). All cell lines were cultured in a humidified atmosphere containing 5% CO_2_ at 37 °C and removed from the plastic substrate using a 0.25% trypsin/EDTA solution for passage and prior to evaluation.

### Intercellular Transport of DOX@BSA from Neutrophils to Tumor Cells

To demonstrate the delivery of DOX@BSA from neutrophils to target tumor cells, 4T1 cells were co‐cultured with the D‐NEs. The 4T1 cells (1 × 10^5^/wells) were seeded in confocal dishes and cultured overnight. Neutrophils were incubated with DOX@BSA with DOX concentration of 5 µg mL^−1^ in a sterile tube containing an FBS‐free RPMI‐1640 medium for 1 h, and free DOX@BSA were washed away via centrifugation. D‐NEs were then added to the co‐culture of 4T1 cells in an FBS‐free medium containing PMA (100 nM) or LPS (5µg mL^−1^). After 8 h of incubation, the cells were washed thrice with ice‐cold PBS, fixed with 4% PFA for 20 min, and stained with DAPI for 5 min. The cells were then examined using CLSM (FLUOVIEW FV1000, Olympus).

### In Vitro Cytotoxicity of PMA‐Triggered D‐NEs Against Tumor Cells

The in vitro cytotoxicity of the D‐NEs against 4T1 and PC3 cells after PMA treatment was measured using the CCK‐8 assay. 4T1 and PC3 cells were seeded in 96‐well plates at a density of 3 × 10^3^ per well and cultured for 24 h. D‐NEs were prepared and pretreated with PMA (100 nM) for 4 h. Subsequently, both untreated and PMA‐treated D‐NEs at different DOX concentrations (0.5, 1, 2, 5, 20, 40 µg m^−1^) were centrifuged at 1400 rpm for 5 min. The supernatant was incubated with 4T1 and PC3 cells for different periods (24 and 48 h). Then, the CCK‐8 solution was added to a final concentration of 10% (VCCK‐8:V1640). After 2 h of incubation, the absorbance was measured at a wavelength of 450 nm using a microplate reader. The relative cell viability was calculated using a previously described method to assess the cytotoxicity of DOX@BSA against neutrophils.

### In Vitro Cytotoxicity of LPS‐Triggered D‐NEs Against Tumor Cells

The in vitro cytotoxicity of the D‐NEs against 4T1, MDA‐MB231, DU145 C4‐2 and PC3 cells after LPS treatment was measured using the CCK‐8 assay. Tumor cells were seeded in 96‐well plates at a density of 3 × 10^3^ per well and cultured for 24 h. D‐NEs were prepared and pretreated with LPS(5µg mL^−1^) for 4 h. Subsequently, LPS‐treated D‐NEs at different DOX concentrations (0.5, 1, 2, 5 µg m^−1^) were centrifuged at 1400 rpm for 5 min. The supernatant was incubated with 4T1 and PC3 cells for 48 h. Then, the CCK‐8 solution was added to a final concentration of 10% (VCCK‐8:V1640). After 2 h of incubation, the absorbance was measured at a wavelength of 450 nm using a microplate reader. The relative cell viability was calculated using a previously described method to assess the cytotoxicity of DOX@BSA against neutrophils.

### Animals and Controllable Inflammation Model

Female BALB/c mice (6–8 weeks, 18–25 g) were purchased from Beijing Si Pei Fu Laboratory Animal Technology Co., Ltd. All the animal experiments were conducted in accordance with the experimental Animal Care and Use guidelines of Tianjin Medical University, China and were approved by the Animal Ethics Committee of the Second Hospital of Tianjin Medical University. The feeding environment was kept clean and hygienic. The temperature was controlled at 25 ± 2 °C, with a 12‐h dark/light cycle and freely available drinking water and food. The animal testing process was guaranteed to be completed under anesthesia. The animals were euthanized before the final dissection. The mice were co‐housed for one week to reduce heterogeneity before being randomly assigned to experimental groups.

To establish a heterotopic tumor model, the mice were given a percutaneous injection of 4T1 cells (3 × 10^6^ cells in 100 µL of PBS per mouse). The tumor growth was monitored daily, and LPS (0.4 mg mL^−1^, 50 µL) was injected intratumorally when the tumor size reached ≈100 mm^3^. The tumor volume was calculated as length × width^2^/2.

### Determination of Infiltration of Neutrophils and Inflammatory Cytokine Expression In Tumor

The infiltration of neutrophils into the tumor after intratumoral injection of LPS was detected using IHE. Tumors were harvested at different times post‐injection (0, 6, 9, 12, and 24 h), followed by immunohistochemical staining with the CD11b antibody. The inflammatory cytokines such as IL‐6, TNF‐*α*, and CXCL1/2 in the tumor after intratumoral injection of LPS‐treated tumor‐bearing mice were measured using an ELISA. The tumors were harvested and weighed at different time points post‐injection and then homogenized in PBS to a concentration of 20% (w:v). The levels of IL‐6, TNF‐*α*, and CXCL1/2 in the brain homogenate were assayed using the corresponding ELISA kits.

### Determination of Controllable Inflammation

Regarding in vivo toxicity, the liver and kidney functions of mice after intratumoral injection of LPS were determined via the blood biochemistry method. LPS (0.4 mg mL^−1^, 50 µL) was injected into the tumors of tumor‐bearing mice, and blood samples were sampled 14 d later. The aspartate (ALT), aspartate aminotransferase (AST), blood urea nitrogen (BUN), and creatinine (Cr) levels were measured using a complete blood panel. For controllable inflammation, 12 h after intratumoral injection of LPS (0.4 mg mL^−1^, 50 µL), different quantities of D‐NEs (5 × 10^5^, 1 × 10^6^, 2 × 10^6^, 4 × 10^6^ cells/mouse) were intravenously injected into the mice, and blood samples were collected 2 h later. Serum was obtained via centrifugation at 8000 rpm for 10 min. The levels of IL‐6 and TNF‐*α* in the serum were assayed using the corresponding ELISA kits.

### In Vivo Tumor‐Targeting Ability of D‐NEs

To evaluate tumor‐targeting capacity of D‐NEs with intratumoral injection of LPS injection, the cell membranes of D‐NEs were first stained with DiD (Beyotime) via incubation with DiD (10 µM) for 10 min. After washing thrice with ice‐cold PBS, DOX@BSA^in^DiD‐NEs (DD‐NEs) were obtained. DD‐NEs (3 × 10^6^ cells/mouse) were intravenously injected into the LPS‐treated and LPS‐untreated tumor‐bearing mice. Subsequently, the mice were imaged 4, 8, 12, 24, and 48 h post‐administration using an IVIS Spectrum imaging system. Thereafter, the mice were sacrificed. The tumors were harvested at predetermined time intervals, and the main organs were collected 12 h post‐administration for ex vivo fluorescence imaging. The ROI was circled around the tumor and organs, and the fluorescence intensity of the DiD signal was analyzed using Living Image Software.

The in vivo biodistribution of DOX delivered by free DOX and D‐NEs, with or without LPS treatment, was determined using an IVIS Spectrum imaging system and CLSM. Briefly, 12 h post‐LPS injection, LPS‐treated tumor‐bearing mice were intravenously injected with free DOX (2.5 mg kg^−1^ DOX) and D‐NEs (3 × 10^6^ cells/mouse, equivalent to 2.5 mg kg^−1^ DOX). In addition, mice in another group were injected intravenously with D‐NEs (3 × 10^6^ cells/mouse, equivalent to 2.5 mg kg^−1^ DOX) without LPS treatment. 24 h after injection, the mice were sacrificed, and the tumors were harvested for ex vivo imaging. Moreover, the tumors were frozen in tissue freezing medium (OCT) at −80 °C and cut into 10‐µm sections later. After staining with DAPI, the slides were visualized using CLSM (FLUOVIEW FV1000, Olympus).

### In Vivo Therapeutic Effect

To verify the induced‐inflammation augmented therapeutic effect of D‐NEs in vivo, 4T1 tumor‐bearing mice were randomly divided into six different groups denoted as the PBS, LPS (0.4 mg mL^−1^, 50 µL), DOX@BSA (2.5 mg kg^−1^ DOX), D‐NEs (3 × 10^6^ cells/mouse), LPS+DOX@BSA (2.5 mg kg^−1^ DOX), and LPS+ D‐NEs (3 × 10^6^ cells/mouse) groups. The mice were given a percutaneous injection of 4T1 cells (3 × 10^6^ cells in 100 µL of PBS per mouse), and LPS was injected intratumorally when the tumor size reached ≈100 mm^3^. At hour 12 and day 3 post‐LPS injection, the tumor‐bearing mice that did or did not receive LPS treatment were separately administrated with the aforementioned six therapies via tail vein injection. The tumor growth, body weights, and survival rates of the mice in each group were monitored and recorded daily.

### Statistical Analysis

Statistical analyses were performed using Prism 8.0 (GraphPad). All the results were expressed as mean ± SD. The Student's t‐test (two‐tailed) was used to evaluate the statistical significance between two groups. For comparisons of statistical significance among more than two groups, an ANOVA was performed. Statistical significance was defined as * *p* < 0.05, ** *p* < 0.01, and *** *p* < 0.001.

## Conflict of Interest

The authors declare no conflict of interest.

## Author Contributions

Y.G., Y.L., and J.L. contributed equally to this work. Y.Z., Y.G., and J.L. performed conceptualization. Y.G., Y.L., and K.L. performed methodology. D.D., W.Z., and H.C. performed investigation. Y.G. performed visualization. G.H., Y.Z., and J.L. performed supervision. Y.G. and Y.L. wrote the original draft. Y.Z. and G.H. wrote, review and edited the final manuscript.

## Supporting information



Supporting Information

## Data Availability

The data that support the findings of this study are available in the supplementary material of this article.
