# Supplementary material for: Controlled Inflammation Drives Neutrophil‐Mediated Precision Drug Delivery in Heterogeneous Tumors
Source: Adv Sci (Weinh). 2025 Jan 12;12(11):2411307. doi: 10.1002/advs.202411307 (PMC11923894; doi:10.1002/advs.202411307)
Supplement: Supplementary file 1 — Supporting Information [file ADVS-12-2411307-s001.pdf]

ADVANCED SCIENCE
Open Access

Supporting Information

for *Adv. Sci.*, DOI 10.1002/advs.202411307

Controlled Inflammation Drives Neutrophil-Mediated Precision Drug Delivery in Heterogeneous Tumors

Yunfei Guo, Yiming Li, Jianmin Li, Haoran Cai, Kangkang Liu, Dengyi Duan, Wenyi Zhang, Gang Han and Yang Zhao**

Supplementary Information

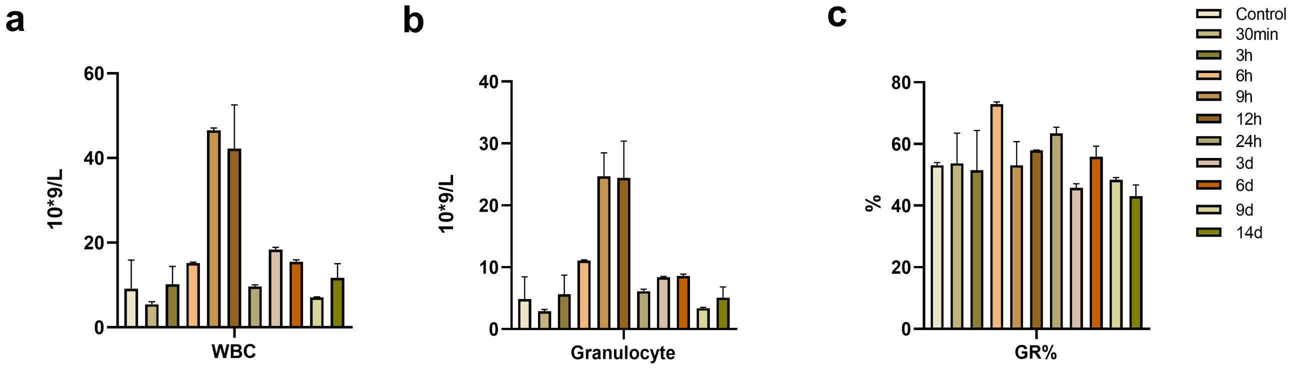

Figure S1. Detection of blood routine indexes after LPS treatment at different time point. a. WBC-white Blood Cell. b. Granulocyte. c. GR%-Granulocyte ratio. (n = 3 mice).

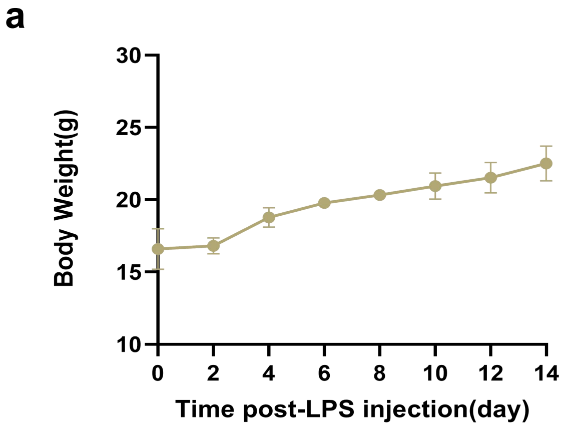

Figure S2. The body weight of mice with LPS treatment over time. (n=3 mice).

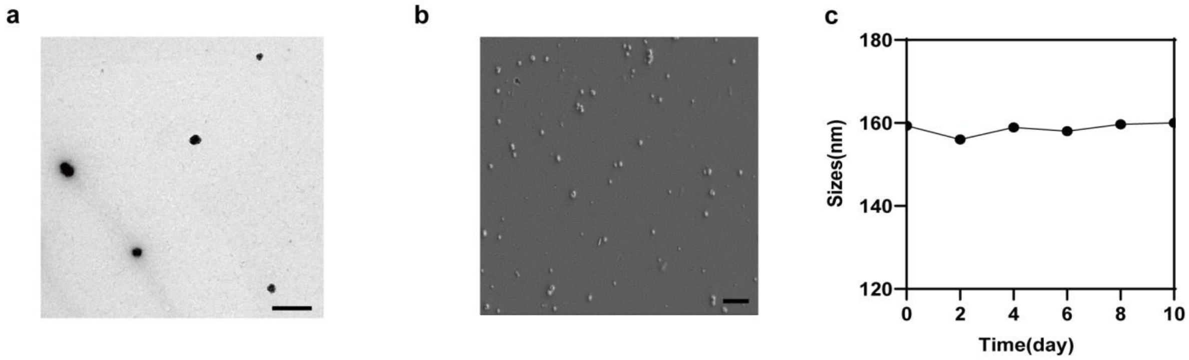

Figure S3. Characterization of DOX@BSA. a. TEM image of DOX@BSA. Scale bar: 500 nm. b. SEM image of DOX@BSA. Scale bar: 2 µm. c. Particle-size changes of DOX@BSA measured by DLS over time.

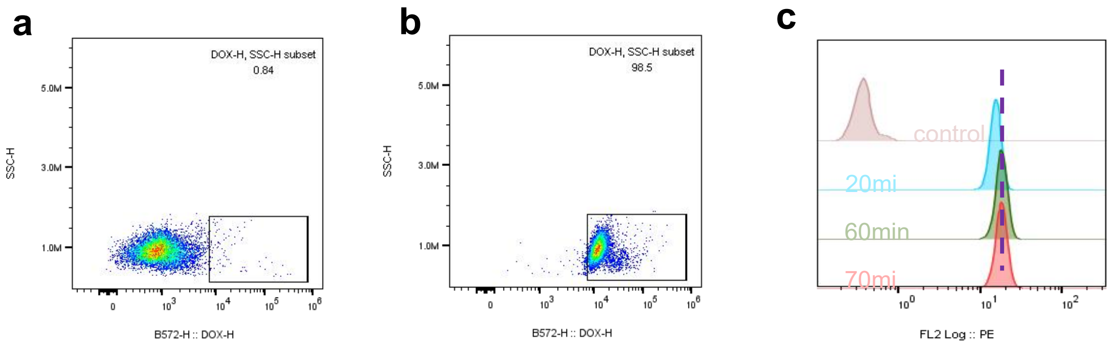

Figure S4. Uptake efficiency of DOX@BSA by neutrophils. a-b. Flow cytometric analysis of neutrophils cultured with medium not containing (a) or containing (b) DOX@BSA for 1 h, respectively. Detection of DOX fluorescence was performed in the PE channel at 488 nm. c. Flow cytometric analysis of neutrophils cultured with DOX@BSA at different time point.

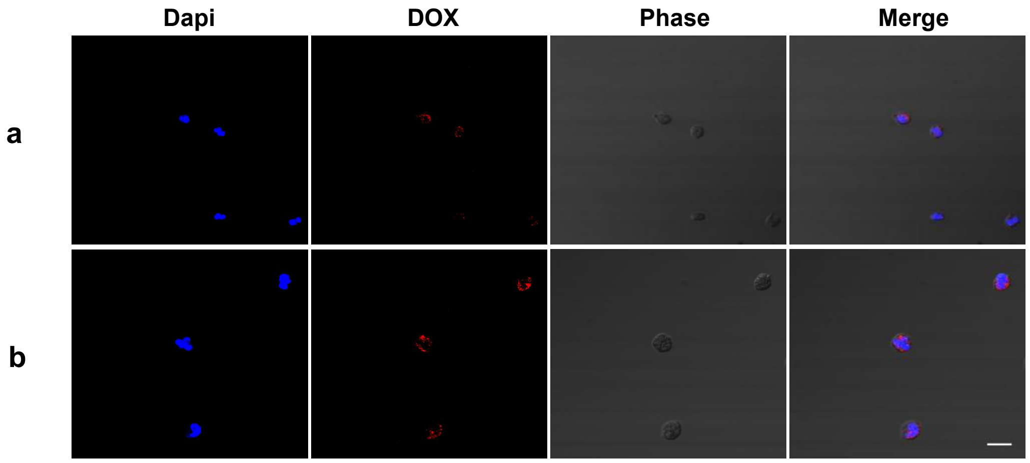

Figure S5. CLSM images of D-NEs. CLSM images of neutrophils incubated with DOX@BSA for 30 min (a) and 1 h (b). The nuclei of neutrophils were labeled with Dapi (blue) and red fluorescence represents DOX encapsulated in the DOX@BSA. The merged image is the overlay of the three individual images. Scale bar: 10 µm.

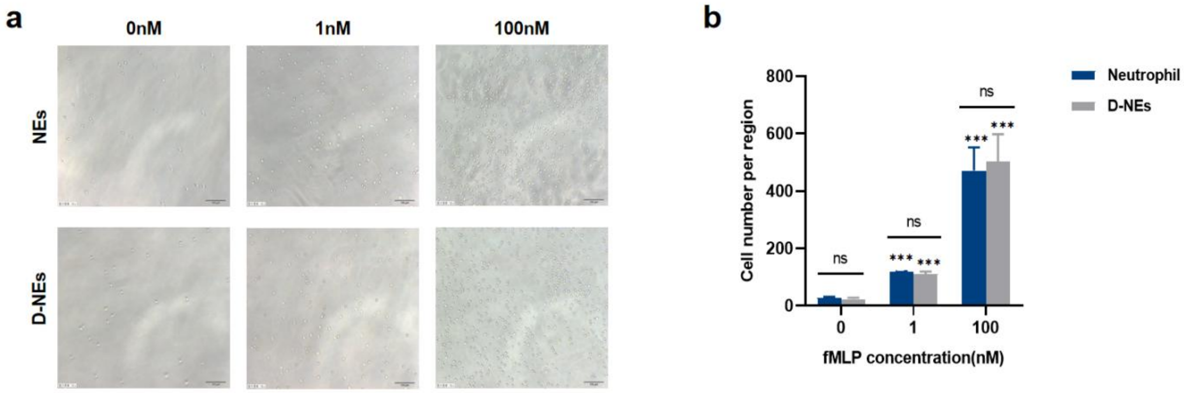

Figure S6. The chemotactic ability of D-NEs. a. Images of blank neutrophils and D-NEs at the lower chamber of the transwell plate after migration test. Scale bar: 100 μm. b. Quantification of migrated neutrophils and D-NEs at the lower chamber of the transwell plate after migration test.

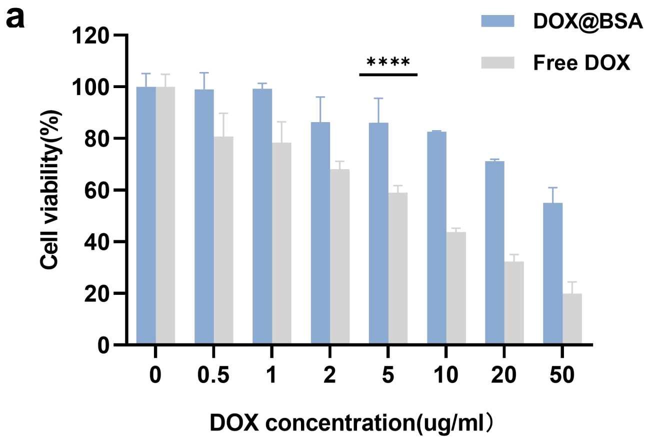

Figure S7. Cytotoxicity of DOX@BSA and free DOX at different DOX concentrations to neutrophils after incubation for 9 h, respectively. (mean ± SD, n = 3 independent experiments, ＊＊＊ $p < 0.001$, ＊＊＊＊ $p < 0.0001$, two-way ANOVA).

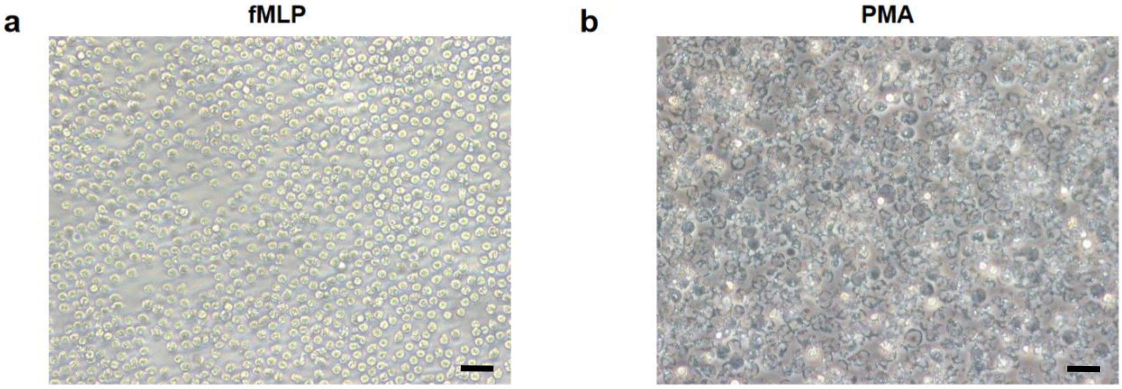

Figure S8. Optical microscope images of D-NEs treated with fMLP (left) or PMA (right) for 6 h. Scale bar: 100 μm

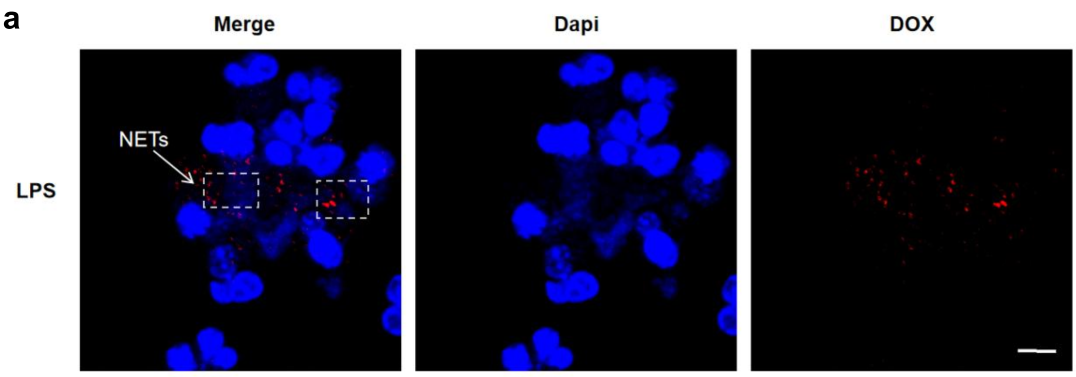

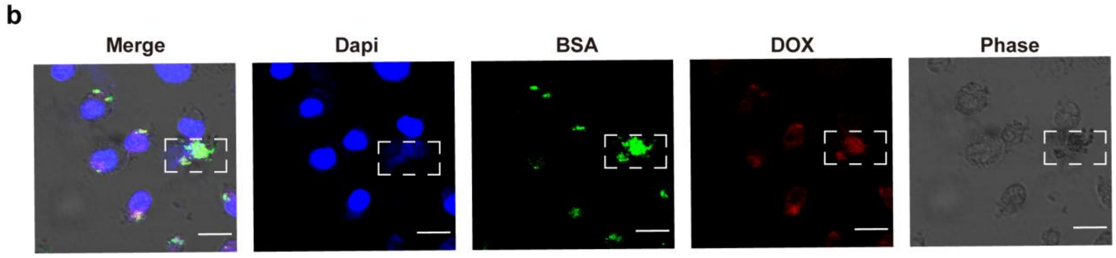

Figure S9. CLSM images of NETs released by D-NEs after 6 h of treatment with LPS. (a).Neutrophil-derived DNA networks were labeled with DAPI (blue), and red fluorescence represents DOX encapsulated in the DOX@BSA. Scale bar: 5 µm. (b). Neutrophil-derived DNA networks were labeled with DAPI (blue), and green fluorescence representsred BSA, and red fluorescence represents DOX encapsulated in the DOX@BSA. Scale bar: 10 µm.

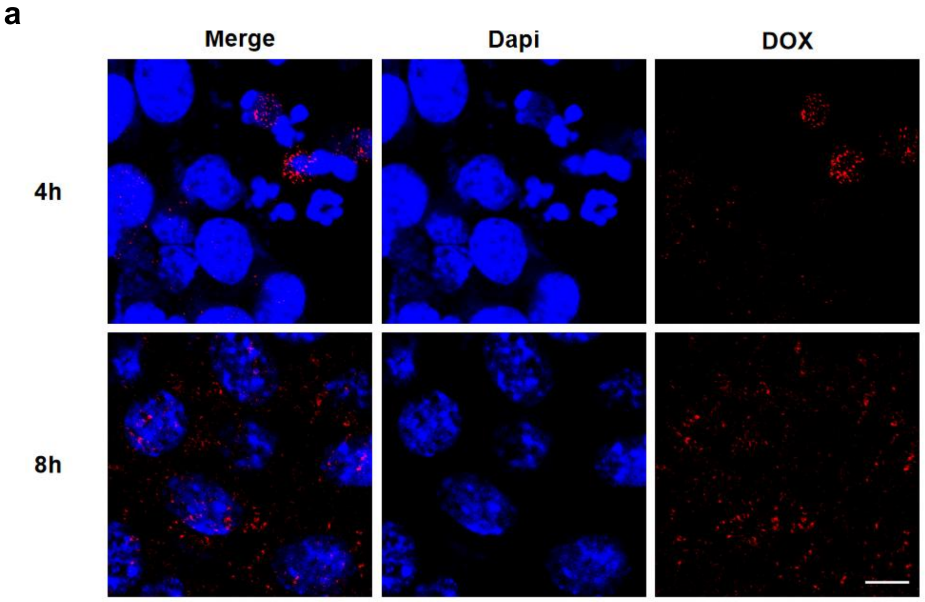

Figure S10. CLSM images of LPS-treated D-NEs incubated with 4T1 cells for 4 and 8 hours. 4T1 cell nuclei and neutrophil-derived DNA networks were labeled with Dapi (blue), and red fluorescence represents DOX encapsulated in the DOX@BSA. Scale bar: 10 µm.

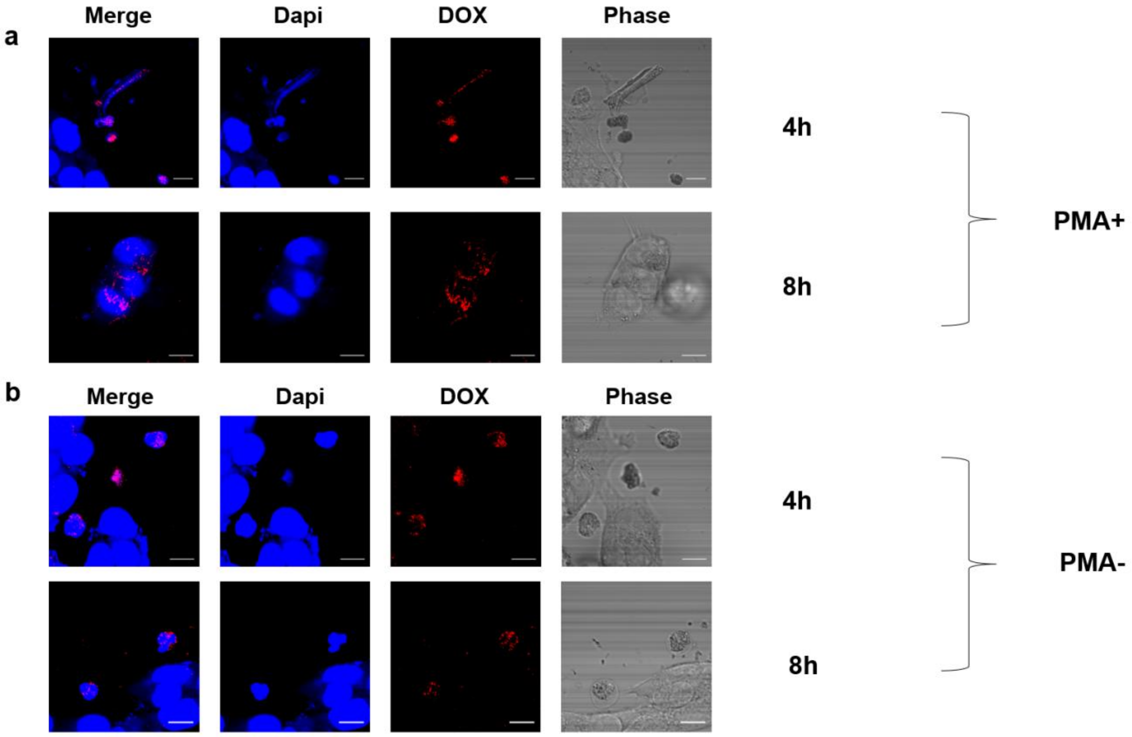

Figure S11. In vitro inflammation-induced drug release. CLSM images of PMA-treated (a) and non-PMA-treated (b) D-NEs incubated with 4T1 cells for 4 and 8 hours. 4T1 cell nuclei and neutrophil-derived DNA networks were labeled with Dapi (blue), and red fluorescence represents DOX encapsulated in the DOX@BSA. Scale bar: 10 μm.

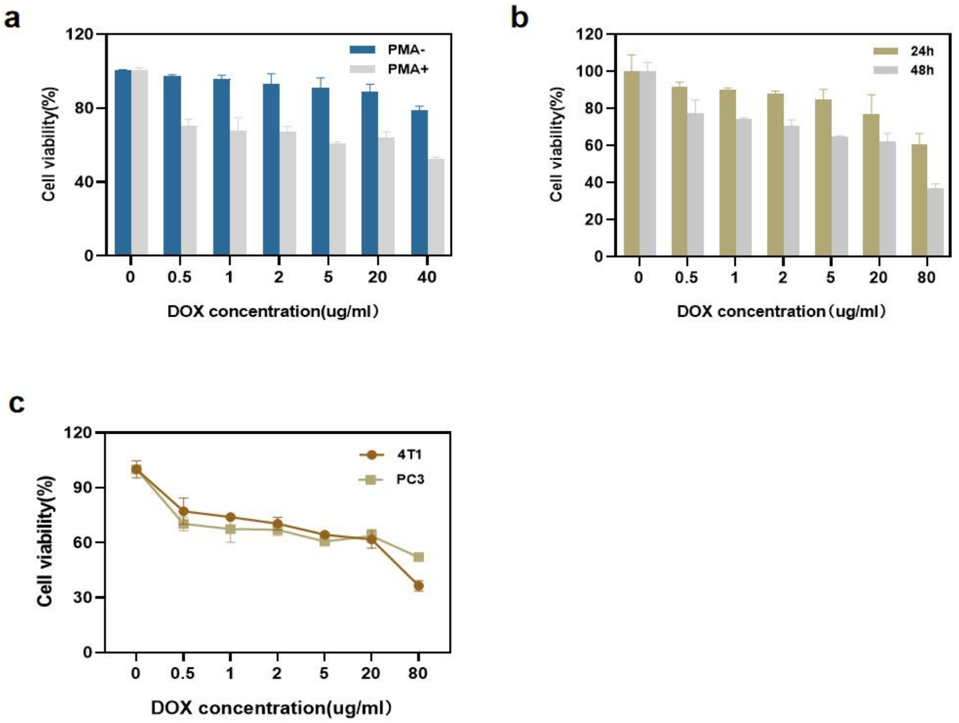

Figure S12. In vitro inflammation-induced tumoricidal effect. a. Cytotoxicity of PMA-treated

D-NEs at different DOX concentrations to PC3 cells. b. Cytotoxicity of PMA-treated D-NEs at different DOX concentrations to 4T1 cells for different times. c. Cytotoxicity of PMA-treated D-NEs at different DOX concentrations against 4T1 cells and PC3 cells.

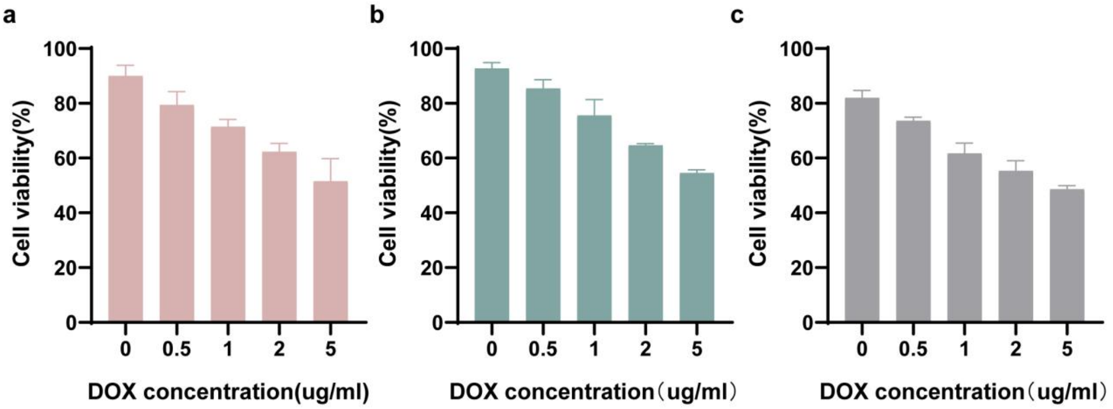

Figure S13. In vitro tumoricidal effect of D-NEs treated with LPS. Cytotoxicity of LPS-treated D-NEs at different DOX concentrations to a. MDA-MB231 cells, b. C4-2 cells, c. DU145 cells.

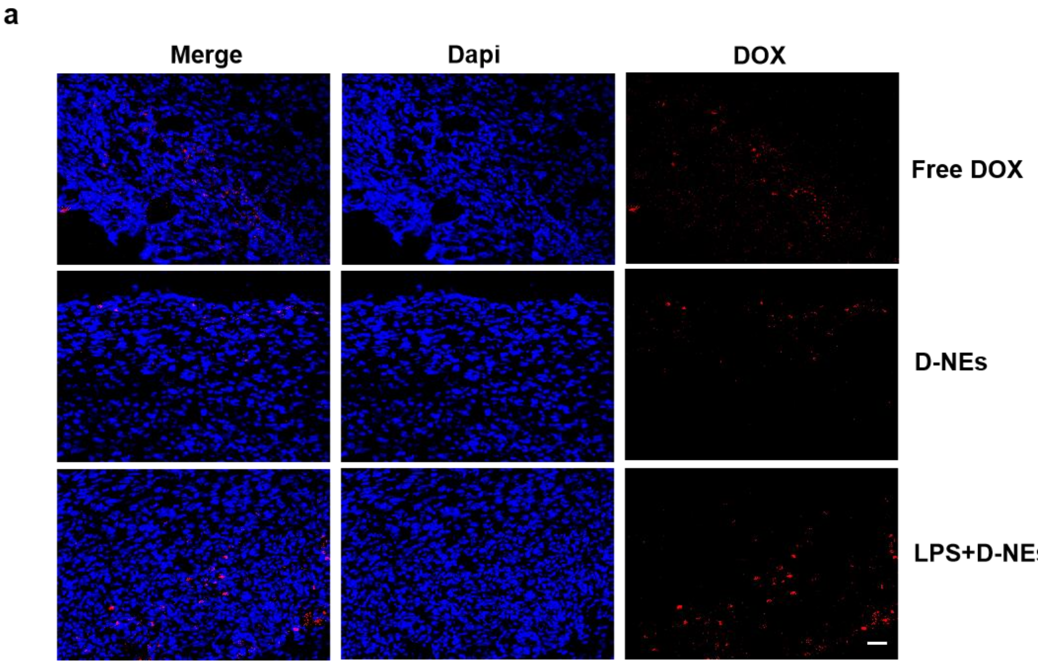

Figure S14. CLSM images of tumors slices excised from mice 12 hours after different treatments. The nuclei of tumor cells were labeled with Dapi (blue), and red fluorescence represents DOX. Scale bar: 30 μm.

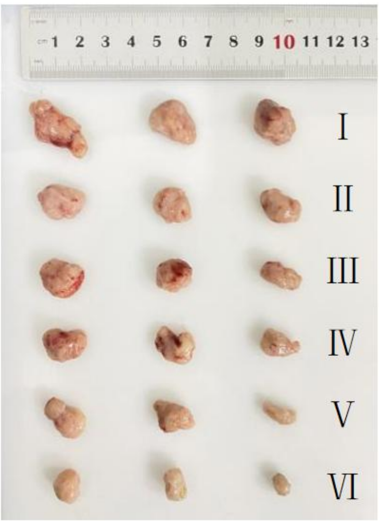

Figure S15. Photographs of extracted tumors removed from 4T1 tumor-bearing mice with different treatments. (I-VI: PBS; LPS; DOX@BSA; D-NEs; LPS+DOX@BSA; LPS+D-NE s).

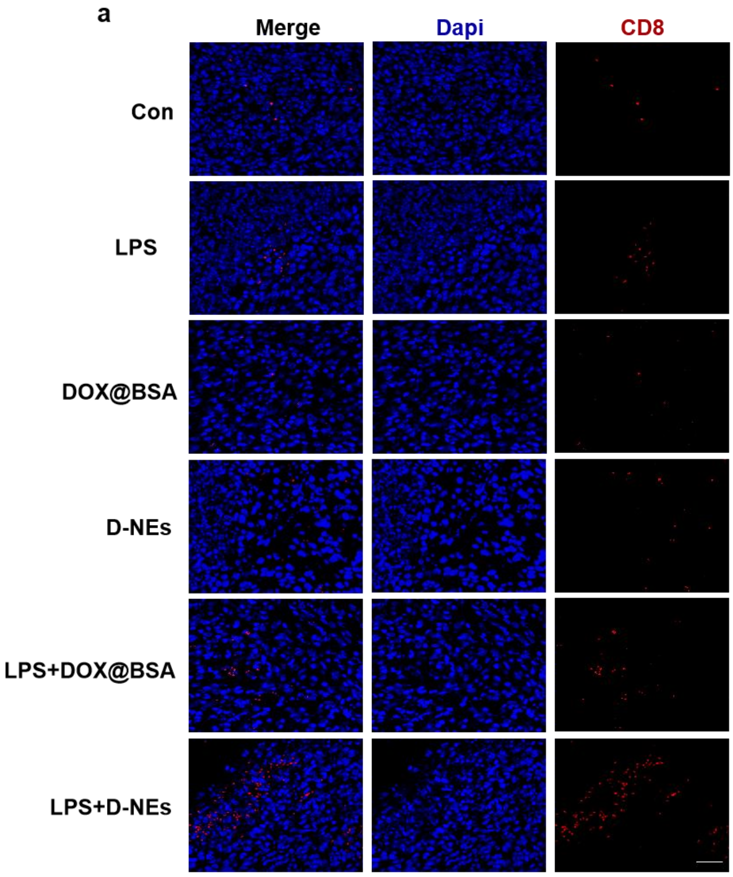

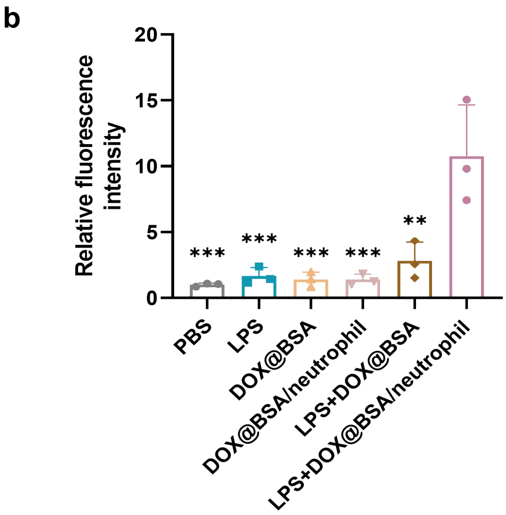

Figure S16. CLSM images of CD8^{+}Tcell in tumor tisssues. a. CLSM images of tumor slices excised from mice after different treatments. The nuclei of tumor cells were labeled with Dapi (blue), and red fluorescence represents CD8. Scale bar: 30 μm.

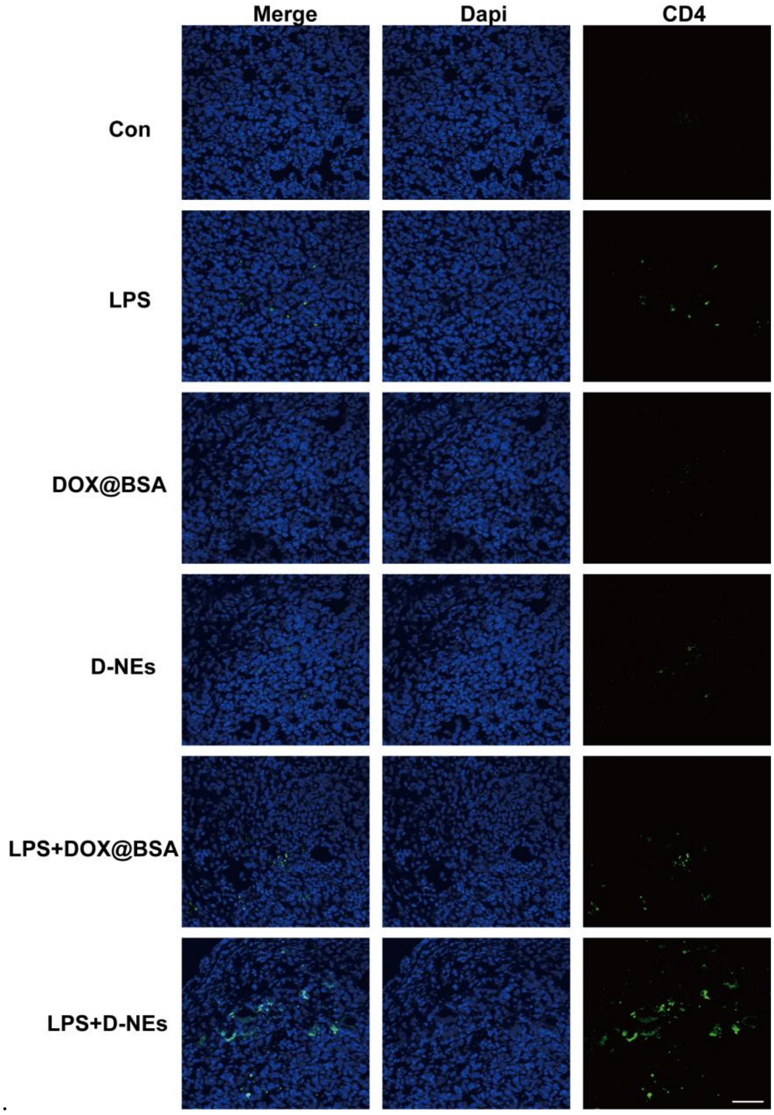

Figure S17. CLSM images of $CD4^{+}$Tcell in tumor tisssues. CLSM images of tumor slices excised from mice after different treatments. The nuclei of tumor cells were labeled with Dapi (blue), and green fluorescence represents CD4. Scale bar: 50 μm

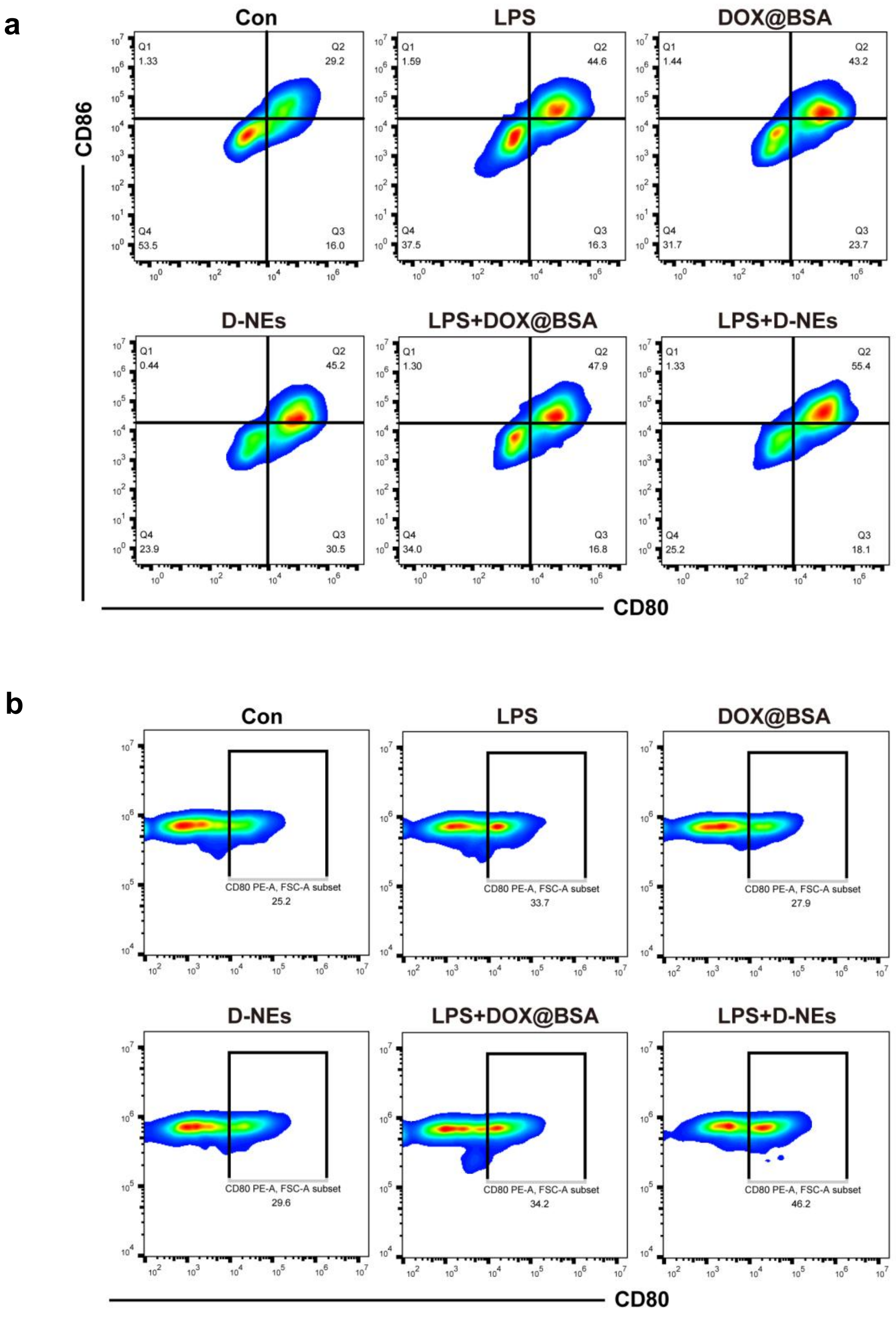

Figure S18. Analysis of dendritic Cells (DCs) and macrophages within tumor tissue. Flow cytometry analysis of (a).CD80+CD86+ Dendritic Cells (DCs), (b).M1 macrophages.

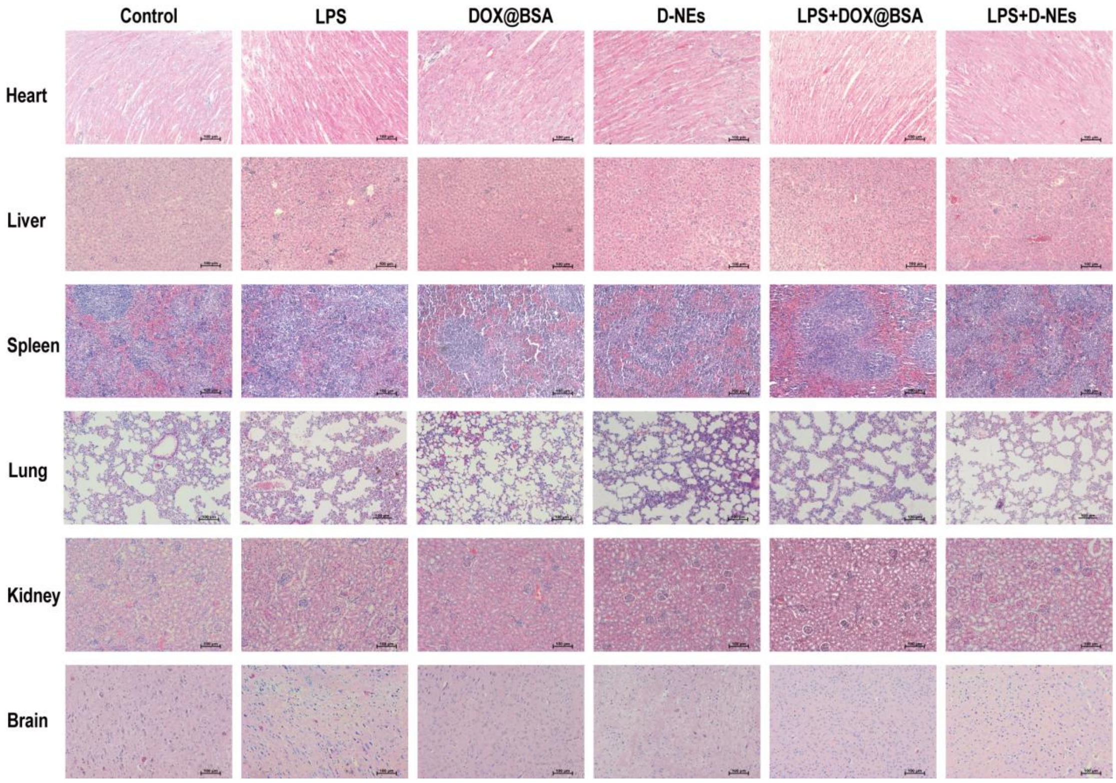

Figure S19. Histological examinations of the heart, liver, spleen, brain, kidney and lung from mice after different treatment. Scale bar: 100μm.

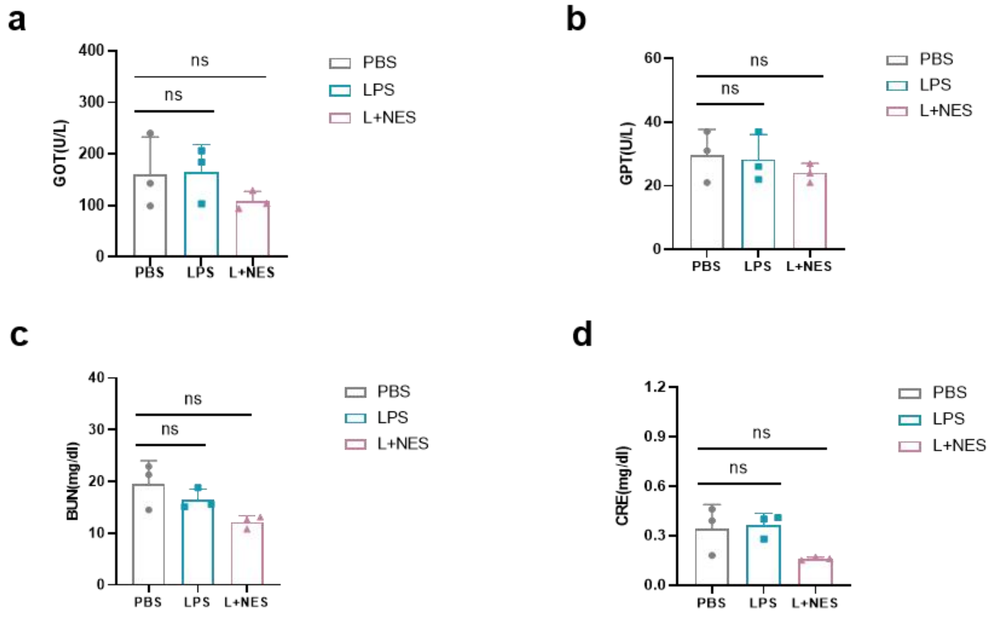

Figure S20. Serological test results after different treatment. PBS was as the control (n = 3 mice).

a

Parameter	Unit	Value											Normal value
		0min	30min	3h	6h	9h	12h	24h	3d	6d	9d	14d	
WBC	10*^{9}/L	5.4	6.0	5.4	15.3	46.0	48.0	9.3	17.8	15.0	7.2	13.5	4.0-19.0
Lymph	10*^{9}/L	1.9	2.7	2.9	1.8	13.7	10.4	2.6	7.2	3.9	2.4	5.2	1.5-18.0
Gran	10*^{9}/L	2.9	2.8	2.0	11.2	23.2	27.8	5.7	8.4	8.7	3.5	6.4	2.0-10.0
Lymph%	%	34.3	44.4	54.0	11.8	29.7	21.7	27.8	40.4	25.6	33.6	38.8	40.0-80.0
Gran%	%	54.0	48.1	36.6	73.4	50.6	58.0	61.9	47.3	58.3	48.9	47.1	24.0-70.0
RBC	10*^{12}/L	6.98	5.5	5.8	6.38	7.29	6.53	7.61	6.12	6.87	6.61	6.08	3.6-13.0
HGB	g/L	162	154	137	159	170	156	188	151	158	163	147	100-190
MCH	pg	23.2	28.0	23.6	24.9	23.3	23.8	24.7	24.6	22.9	24.6	24.1	16.0-24.0
RDW-SD	fL	26.0	26.0	26.0	26.0	26.0	26.0	26.0	26.0	26.0	26.0	27.9	37.0-54.0
RDW-CV	%	13.5	14.3	13.6	14.0	13.3	13.5	13.5	13.5	13.5	13.4	14.6	11.0-15.5
PLT	10*^{9}/L	271	174	67	137	84	143	94	378	472	207	155	300-600
MPV	fL	8.6	9.2	7.4	7.4	10.1	9.3	7.8	9.0	9.1	9.2	8.6	7.0-12.0

Table S1. Detection of blood routine indexes after LPS treatment at different time point.
